# Comparative effectiveness of decompressive craniectomy versus craniotomy for traumatic acute subdural hematoma (CENTER-TBI): an observational cohort study

**DOI:** 10.1016/j.eclinm.2023.102161

**Published:** 2023-08-09

**Authors:** Thomas A. van Essen, Inge A.M. van Erp, Hester F. Lingsma, Dana Pisică, John K. Yue, Ranjit D. Singh, Jeroen T.J.M. van Dijck, Victor Volovici, Alexander Younsi, Angelos Kolias, Lianne D. Peppel, Majanka Heijenbrok-Kal, Gerard M. Ribbers, David K. Menon, Peter J.A. Hutchinson, Geoffrey T. Manley, Bart Depreitere, Ewout W. Steyerberg, Andrew I.R. Maas, Godard C.W. de Ruiter, Wilco C. Peul, CENTER-TBI Investigators and Participants, Cecilia Åkerlund, Krisztina Amrein, Nada Andelic, Lasse Andreassen, Audny Anke, Anna Antoni, Gérard Audibert, Philippe Azouvi, Maria Luisa Azzolini, Ronald Bartels, Pál Barzó, Romuald Beauvais, Ronny Beer, Bo-Michael Bellander, Antonio Belli, Habib Benali, Maurizio Berardino, Luigi Beretta, Morten Blaabjerg, Peter Bragge, Alexandra Brazinova, Vibeke Brinck, Joanne Brooker, Camilla Brorsson, Andras Buki, Monika Bullinger, Manuel Cabeleira, Alessio Caccioppola, Emiliana Calappi, Maria Rosa Calvi, Peter Cameron, Guillermo Carbayo Lozano, Marco Carbonara, Ana M. Castaño-León, Simona Cavallo, Giorgio Chevallard, Arturo Chieregato, Giuseppe Citerio, Hans Clusmann, Mark Steven Coburn, Jonathan Coles, Jamie D. Cooper, Marta Correia, Amra Čović, Nicola Curry, Endre Czeiter, Marek Czosnyka, Claire Dahyot-Fizelier, Paul Dark, Helen Dawes, Véronique De Keyser, Vincent Degos, Francesco Della Corte, Hugo den Boogert, Bart Depreitere, Đula Đilvesi, Abhishek Dixit, Emma Donoghue, Jens Dreier, Guy-Loup Dulière, Ari Ercole, Patrick Esser, Erzsébet Ezer, Martin Fabricius, Valery L. Feigin, Kelly Foks, Shirin Frisvold, Alex Furmanov, Pablo Gagliardo, Damien Galanaud, Dashiell Gantner, Guoyi Gao, Pradeep George, Alexandre Ghuysen, Lelde Giga, Ben Glocker, Jagoš Golubović, Pedro A. Gomez, Johannes Gratz, Benjamin Gravesteijn, Francesca Grossi, Russell L. Gruen, Deepak Gupta, Juanita A. Haagsma, Iain Haitsma, Raimund Helbok, Eirik Helseth, Lindsay Horton, Jilske Huijben, Peter J. Hutchinson, Bram Jacobs, Stefan Jankowski, Mike Jarrett, Ji-yao Jiang, Faye Johnson, Kelly Jones, Mladen Karan, Angelos G. Kolias, Erwin Kompanje, Daniel Kondziella, Evgenios Kornaropoulos, Lars-Owe Koskinen, Noémi Kovács, Alfonso Lagares, Linda Lanyon, Steven Laureys, Fiona Lecky, Didier Ledoux, Rolf Lefering, Valerie Legrand, Aurelie Lejeune, Leon Levi, Roger Lightfoot, Hester Lingsma, Andrew I.R. Maas, Marc Maegele, Marek Majdan, Alex Manara, Geoffrey Manley, Hugues Maréchal, Costanza Martino, Julia Mattern, Catherine McMahon, Béla Melegh, David Menon, Tomas Menovsky, Ana Mikolic, Benoit Misset, Visakh Muraleedharan, Lynnette Murray, Nandesh Nair, Ancuta Negru, David Nelson, Virginia Newcombe, Daan Nieboer, József Nyirádi, Matej Oresic, Fabrizio Ortolano, Olubukola Otesile, Aarno Palotie, Paul M. Parizel, Jean-François Payen, Natascha Perera, Vincent Perlbarg, Paolo Persona, Wilco Peul, Anna Piippo-Karjalainen, Matti Pirinen, Dana Pisica, Horia Ples, Suzanne Polinder, Inigo Pomposo, Jussi P. Posti, Louis Puybasset, Andreea Rădoi, Arminas Ragauskas, Rahul Raj, Malinka Rambadagalla, Veronika Rehorčíková, Isabel Retel Helmrich, Jonathan Rhodes, Sylvia Richardson, Sophie Richter, Samuli Ripatti, Saulius Rocka, Cecilie Roe, Olav Roise, Jonathan Rosand, Jeffrey Rosenfeld, Christina Rosenlund, Guy Rosenthal, Rolf Rossaint, Sandra Rossi, Daniel Rueckert, Martin Rusnák, Juan Sahuquillo, Oliver Sakowitz, Renan Sanchez-Porras, Janos Sandor, Nadine Schäfer, Silke Schmidt, Herbert Schoechl, Guus Schoonman, Rico Frederik Schou, Elisabeth Schwendenwein, Charlie Sewalt, Toril Skandsen, Peter Smielewski, Abayomi Sorinola, Emmanuel Stamatakis, Simon Stanworth, Ana Kowark, Robert Stevens, William Stewart, Ewout W. Steyerberg, Nino Stocchetti, Nina Sundström, Riikka Takala, Viktória Tamás, Tomas Tamosuitis, Mark Steven Taylor, Braden Te Ao, Olli Tenovuo, Alice Theadom, Matt Thomas, Dick Tibboel, Marjolijn Timmers, Christos Tolias, Tony Trapani, Cristina Maria Tudora, Andreas Unterberg, Peter Vajkoczy, Egils Valeinis, Shirley Vallance, Zoltán Vámos, Mathieu Van der Jagt, Joukje van der Naalt, Gregory Van der Steen, Jeroen T.J.M. van Dijck, Thomas A. van Essen, Wim Van Hecke, Caroline van Heugten, Dominique Van Praag, Ernest Van Veen, Roel van Wijk, Thijs Vande Vyvere, Alessia Vargiolu, Emmanuel Vega, Kimberley Velt, Jan Verheyden, Paul M. Vespa, Anne Vik, Rimantas Vilcinis, Victor Volovici, Nicole von Steinbüchel, Daphne Voormolen, Petar Vulekovic, Kevin K.W. Wang, Eveline Wiegers, Guy Williams, Lindsay Wilson, Stefan Winzeck, Stefan Wolf, Zhihui Yang, Peter Ylén, Alexander Younsi, Frederick A. Zeiler, Agate Ziverte, Tommaso Zoerle

**Affiliations:** a University Neurosurgical Center Holland, Leiden University Medical Center, Haaglanden Medical Center, HAGA, Leiden and The Hague, the Netherlands; b Division of Neurosurgery, Department of Clinical Neurosciences, University of Cambridge and Addenbrooke's Hospital, Cambridge, United Kingdom; c Department of Surgery, Division of Neurosurgery, QEII Health Sciences Centre and Dalhousie University, Halifax, Nova Scotia, Canada; d Center for Medical Decision Making, Department of Public Health, Erasmus MC - University Medical Center, Rotterdam, the Netherlands; e Department of Neurosurgery, Erasmus MC – University Medical Center, Rotterdam, the Netherlands; f Brain and Spinal Injury Center, Department of Neurological Surgery, Zuckerberg San Francisco General Hospital, University of California San Francisco, San Francisco, USA; g Department of Neurosurgery, University Hospital Heidelberg, University of Heidelberg, Heidelberg, Germany; h NIHR Global Health Research Group on Neurotrauma, University of Cambridge, Cambridge, United Kingdom; i Rijndam Rehabilitation and Department of Rehabilitation Medicine, Erasmus MC – University Medical Center, Rotterdam, the Netherlands; j Division of Anaesthesia, Addenbrooke's Hospital, University of Cambridge, Cambridge, United Kingdom; k Department of Neurosurgery, University Hospital KU Leuven, Leuven, Belgium; l Department of Biomedical Data Sciences, Leiden University Medical Center, Leiden, the Netherlands; m Department of Neurosurgery, Antwerp University Hospital and University of Antwerp, Edegem, Belgium

**Keywords:** Acute subdural hematoma, Decompressive craniectomy, Craniotomy, Comparative effectiveness research, Instrumental variable analysis, Practice variation

## Abstract

**Background:**

Limited evidence existed on the comparative effectiveness of decompressive craniectomy (DC) versus craniotomy for evacuation of traumatic acute subdural hematoma (ASDH) until the recently published randomised clinical trial RESCUE-ASDH. In this study, that ran concurrently, we aimed to determine current practice patterns and compare outcomes of primary DC versus craniotomy.

**Methods:**

We conducted an analysis of centre treatment preference within the prospective, multicentre, observational Collaborative European NeuroTrauma Effectiveness Research in Traumatic Brain Injury (known as CENTER-TBI) and NeuroTraumatology Quality Registry (known as Net-QuRe) studies, which enrolled patients throughout Europe and Israel (2014–2020). We included patients with an ASDH who underwent acute neurosurgical evacuation. Patients with severe pre-existing neurological disorders were excluded. In an instrumental variable analysis, we compared outcomes between centres according to treatment preference, measured by the case-mix adjusted proportion DC per centre. The primary outcome was functional outcome rated by the 6-months Glasgow Outcome Scale Extended, estimated with ordinal regression as a common odds ratio (OR), adjusted for prespecified confounders. Variation in centre preference was quantified with the median odds ratio (MOR). CENTER-TBI is registered with ClinicalTrials.gov, number NCT02210221, and the Resource Identification Portal (Research Resource Identifier SCR_015582).

**Findings:**

Between December 19, 2014 and December 17, 2017, 4559 patients with traumatic brain injury were enrolled in CENTER-TBI of whom 336 (7%) underwent acute surgery for ASDH evacuation; 91 (27%) underwent DC and 245 (63%) craniotomy. The proportion primary DC within total acute surgery cases ranged from 6 to 67% with an interquartile range (IQR) of 12–26% among 46 centres; the odds of receiving a DC for prognostically similar patients in one centre versus another randomly selected centre were trebled (adjusted median odds ratio 2.7, *p* < 0.0001). Higher centre preference for DC over craniotomy was not associated with better functional outcome (adjusted common odds ratio (OR) per 14% [IQR increase] more DC in a centre = 0.9 [95% CI 0.7–1.1], n = 200). Primary DC was associated with more follow-on surgeries and complications [secondary cranial surgery 27% vs. 18%; shunts 11 vs. 5%]; and similar odds of in-hospital mortality (adjusted OR per 14% IQR more primary DC 1.3 [95% CI (1.0–3.4), n = 200]).

**Interpretation:**

We found substantial practice variation in the employment of DC over craniotomy for ASDH. This variation in treatment strategy did not result in different functional outcome. These findings suggest that primary DC should be restricted to salvageable patients in whom immediate replacement of the bone flap is not possible due to intraoperative brain swelling.

**Funding:**

10.13039/501100008358Hersenstichting Nederland for the Dutch NeuroTraumatology Quality Registry and the European Union Seventh Framework Program.

**Research in context**

**Evidence before this study**

In preparation of these comparative effectiveness studies, we systematically reviewed the evidence on the surgical approaches for acute subdural hematoma (ASDH). The protocols of this assessment are available online (PROSPERO registration numbers CRD42015025491 and CRD42019125336). We searched English and Dutch publications in the databases IndexCAT, PubMed, Embase (OVID-version), Web of Science, Cochrane library, CENTRAL, Academic Search Premier, Google Scholar, ScienceDirect, and CINAHL. The search string focused on traumatic ASDH, cranial surgery, and outcome, and was devised with a trained librarian. This initial search was completed in November 24, 2021, and the first results were published in January 2023. After risk of bias evaluation, no comparative studies with a low risk of bias were found. RESCUE-ASDH was published after this period.

This study runs parallel to the recently published RESCUE-ASDH randomised clinical trial (RCT) in which the conclusion was that primary decompressive craniectomy (DC) and craniotomy lead to similar functional outcome and quality of life. The best-known guidelines on surgery for acute subdural hematoma (ASDH), from the Brain Trauma Foundation, were published in 2006 and consisted of a review of the available studies. The conclusions (for ASDH surgery) were based on the lowest grade on the effectiveness evidence hierarchy; at best, retrospective observational studies. Since then, no update has been published. In July 2022, we published a comparative effectiveness study on surgery versus conservative treatment for ASDH. No difference was found between surgery and conservative treatment. The current study is a (predefined) analysis of the surgical arm of this comparative effectiveness study.

**Added value of this study**

We report on the largest observational comparative effectiveness study of surgery in ASDH. The results are in line with those from RESCUE-ASDH and extend outside the controlled setting of the RCT. It was performed across multiple centres in Europe, and thus generalisable to a broad population. We found substantial practice variation in the use of DC for ASDH, reflecting the lack of strong evidence (up to RESCUE-ASDH). In an instrumental variable analysis, this variation in primary DC did not result in differences in outcome for DC versus craniotomy. Our study corroborates the findings of RESCUE-ASDH, provides real-world evidence that is generalisable to a broad population and thereby, ensures safe application in routine clinical practice.

**Implications of all the available evidence**

The large practice variation among trauma centres is a reflection of the uncertainty among neurosurgeons as to whether to perform a pre-emptive DC when operating on a patient with an ASDH. By exploiting this strong and consistent treatment preference, our study provides a real-world estimate of effectiveness for those patients with ASDH for whom equipoise exists on indication for primary DC. Furthermore, the large variation in primary DC rates across Europe attests to the potential improvement by implementing the findings of RESCUE-ASDH and this study. The low threshold employed for pre-emptive primary DC cannot be upheld anymore. Our study also shows the potential validity of observational evidence in determining treatment effectiveness. High-quality data collection and sophisticated analysis can lead to unbiased estimates for acute neurosurgical decisions, even in the presence of strong confounding. We acknowledge, however, that potential differences in effect estimates would not necessarily mean that observational results would be invalid. Differences could be due to different methodological choices and, indeed, also bias. Thus, our current study could act as an impetus to start bring evidence-based consistency to current practice to get better overall outcomes for patients with an ASDH in need of surgery.

## Introduction

Acute subdural hematomas (ASDH) present in approximately one-third of patients with severe traumatic brain injury (TBI).^1^^,^^2^ This space-occupying hematoma, can severely reduce blood flow to the brain, and elevate intracranial pressure (ICP), causing brain herniation, poor functional outcome, and death.^3^ The decision to treat a patient surgically or conservatively in the acute phase turns on their neurological status, the size of the hematoma, and the degree of mass effect.^4^

Surgical procedure to evacuate ASDH follows one of two approaches: craniotomy with reconstruction of the skull with the bone flap replaced, or decompressive craniectomy (DC), in which the bone flap is not immediately rebuilt to mitigate (future) ICP increase. Several clinical scenarios guide the surgical decision. Primary DC is performed if, after ASDH evacuation, the brain swells beyond the skull intraoperatively, preventing safe replacement of the flap without pathological ICP rise. Another scenario is preventive, if there is concern that the brain may swell post-operatively.^5^ Secondary DC is performed later in the clinical course, as a last-resort after exhaustion of neurocritical care measures, with clear benefits to functional outcomes.^6^

DC is considered more invasive than craniotomy, as it leads to a temporary bone defect, requires later skull reconstruction (cranioplasty), and is associated with greater occurrence of post-traumatic hydrocephalus, bone flap reabsorption, and post-cranioplasty infection.^7^ The Brain Trauma Foundation guideline for surgical treatment of ASDH provides no clear indication for selection of approach.^8^

In April 2023 the RESCUE-ASDH (Randomized Evaluation of Surgery with Craniectomy for Patients Undergoing Evacuation of Acute Subdural Hematoma) RCT was published that compared both approaches.^9^ The conclusion was that primary DC and craniotomy lead to similar functional outcome and quality of life. The other literature analysing selection of technique has methodologic limitations, and comes largely from retrospective cohort studies.^1^^,^^4^^,^^10^^,^^11^ This lack of high-quality evidence up to the publication of RESCUE-ASDH, may have led to practice variation comparing neurosurgical centers.^12^^,^^13^ Comparative-effectiveness research (CER) can exploit this variation to determine optimal management and provides real-world evidence.^14^ In this observational study, conducted parallel to RESCUE-ASDH, we first aimed to determine current treatment practices. Second, we compared primary DC versus craniotomy for ASDH for functional outcome at 6 months.

## Methods

### Study design

This is a prospective, observational, cohort study within the Collaborative European NeuroTrauma Effectiveness Research in TBI (CENTER-TBI), which enrolled patients between 2014 and 2017 in 65 centres across Europe and Israel.^o^^,^^15^^,^^16^ Parent studies were conducted in accordance with Good Clinical Practice (CPMP/ICH/135/95).

The study was predefined in a protocol with the hypothesis of better outcomes for primary DC.^17^ The hypothesis is based on the evidence up to RESCUE-ASDH.^18^^,^^19^ The study and predefined protocol follow the Strengthening the Reporting of Observational Studies in Epidemiology statement with instrumental variable (IV) analyses recommendations, and corresponds to stage 3 in the IDEAL framework.^17^^,^^20^ The study was planned to use the convenience sample provided by CENTER-TBI. CENTER-TBI is registered with ClinicalTrials.gov (number NCT02210221), and the Resource Identification Portal (Research Resource Identifier SCR_015582).

### Ethics

The CENTER-TBI study was approved by the medical ethics committees of all participating centres. Informed written or oral consent by patients or legal representatives was obtained according to local regulation.

### Study population/data management

The CENTER-TBI cohort included patients with TBI and no pre-existing severe neurological disorders that could affect outcome assessment, who presented within 24 h of trauma, and who had a brain CT ordered as part of clinical care. For the current study, we selected patients from the CENTER-TBI cohort with an ASDH confirmed on admission CT who received acute surgery.^2^ We excluded patients who received a craniotomy for other types of injury, those that were brain dead, and those considered by the treating doctor to have an unsurvivable injury, for whom active treatment was futile. Data were collected by trained personnel using online case-report forms (QuesGen Systems, Burlingame, CA, USA), coded with the NIH-NINDS Common Data Elements.^15^

### Centre characteristics

Centre characteristics have been previously reported.^12^ Questions included centre policy regarding the threshold for primary DC, which was used in sensitivity analyses. Other treatment decisions possibly related to surgical threshold (e.g., prehospital care) could affect the internal validity of the study. We therefore did a cluster analysis, showing that centre surgical treatment preferences were unrelated to other treatment preferences.^21^

### Interventions

Acute hematoma evacuation was performed via craniotomy or primary DC, at the discretion of the treating neurosurgeon. Treatment groups were classified according to first (presenting) CT. Per study protocol, neurosurgeons were queried as to reason(s) surgery was indicated, surgical approach, and confirmed according to operating room disposition and by intervention codes or description. Techniques for durotomy and potential duroplasty were not routinely collected. Other emergency and post-surgical care followed local protocols (ICU management, ICP monitoring, and/or follow-on surgery).

### Outcomes

The primary outcome was functional outcome at 6 months on the Glasgow Outcome Scale-Extended (GOSE).^22^ Secondary outcomes were in-hospital mortality, ICP, frequency and type of neurosurgical interventions, medical and surgical complications, ‘treatment failure’ (subsequent craniotomy or DC), ICU and hospital length of stay (days), dichotomised 6-month GOSE score across multiple thresholds, and quality of life at 6-months postinjury, measured with the Quality of Life after Brain Injury instrument (QOLIBRI).^23^

### Statistical analysis

Baseline characteristics are presented using descriptive statistics, including standardised mean differences across the instrument and between groups. The CRASH-CT head injury model was used to calculate predicted probabilities of unfavourable outcome.^24^ We calculated the median odds ratio (MOR) to compare between-centre differences in surgery. The MOR quantifies treatment variation between centres that is not attributable to chance and not explained by other (case-mix) factors.

Outcomes were analysed with respect to centre treatment strategy (and not actual treatment) using instrumental variable (IV) analyses. In this natural experiment the IV “allocates” patients to either the DC or craniotomy treatment strategy based on the treating centre, and reduces (unmeasured) confounding (Appendix 12–13).

The common odds ratio (OR) was estimated with a random-effects multivariable proportional odds logistic regression model with the ordinal GOSE as outcome variable, the case-mix adjusted centre-specific treatment probability of DC as the independent variable (the IV), and a random intercept for treating centre (unexplained residual between-center differences). The OR summarises the shift in the direction of a better score on the GOSE. Adjustment was made for age, GCS, pupillary reactivity, midline shift, concomitant contusion, and hematoma size as potential confounders. The resulting adjusted common OR was presented as an increase from the first to the fourth quartile (IQR) of the (continuous) instrumental variable (the adjusted probabilities for undergoing DC) and can be interpreted as the odds of a more favourable outcome when comparing centres favouring a strategy of primary DC versus those favouring craniotomies. We excluded centres that provided <10 patients for the primary analysis to minimise the influence of chance findings.

Sensitivity analysis included IV analysis using centre-preference for primary DC as the instrumental variable, per prior published provider profile.^12^ Sensitivity IV analysis was also performed excluding centres with <15 patients (as opposed to <10 patients of the primary analysis). Furthermore, the IV association of surgical preference with outcome was also estimated by linear regression with the case-mix adjusted probability of DC (treatment preference) as the independent variable, mean GOSE by centre as the dependent variable, and similar adjustment. Last, the main analysis was repeated post-hoc on patients without poor baseline prognoses, which was set at a CRASH-CT score within the fourth quartile (ie, higher than 85% predicted unfavorable outcome [proportion with a Glasgow Outcome Scale score ≤3]).

We performed unadjusted and multivariable regression and propensity score matching (PSM) as sensitivity analyses with actual DC received as treatment variable (yes/no; not centre DC preference) and GOSE as ordinal outcome variable. We determined adjusted ORs (aOR) for multiple cut-off values on the GOSE to assess consistency of effect estimates. Further details are supplemented (Appendix 12–13). Analyses were conducted using R-software 4.1.0, RStudio 1.1.463. Missing data were multiply imputed (‘mice’ package, m = 5), assuming data to be missing-at-random. The 95% CIs for the ORs were obtained from 2.5 to 97.5 percentiles among the bootstrap replications.

### Role of the funding source

The funders of the study had no role in study design, data collection, data analysis, data interpretation, or writing of the report.

## Results

**Fig. 1:**
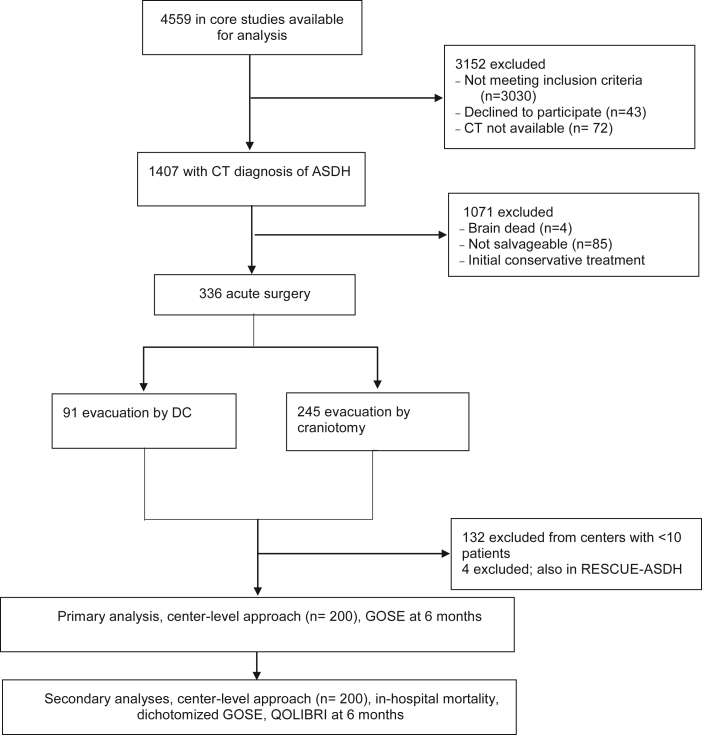
**Flow diagram of study population and data analyses**. DC, decompressive craniectomy; GOSE, Glasgow Outcome Scale Extended; QOLIBRI, Quality of Life after Brain Injury Questionnaire; RESCUE-ASDH, Randomized Evaluation of Surgery with Craniectomy for Patients Undergoing Evacuation of Acute Subdural Hematoma.

Between December 19, 2014 and December 17, 2017, 4559 patients with traumatic brain injury were enrolled in CENTER-TBI. 336 of 4559 patients underwent acute surgery for an ASDH, of whom 91 (27%) received a primary DC and 245 (73%) received a craniotomy (Fig. 1). Median time from injury to start of surgery was 3.5 h for primary DC (IQR 2.2–5.1) and 4.1 h for craniotomy (IQR 2.8–7.0). Patients undergoing primary DC were younger (median age 49 vs. 59 years), less often on anticoagulants and/or platelet aggregation inhibitors (14 vs. 25%), with more major extracranial injuries (53 vs. 36%), worse presenting GCS scores (median 4 vs. 7), larger ASDH volumes (median 64 vs. 49 cm^3^), and more frequent contusions and subarachnoid haemorrhages (66% vs. 55%, and 75% vs. 62% respectively; Appendix 15–17).

These baseline characteristics did not translate into different predicted 6-month unfavourable outcomes calculated according to the CRASH-CT for primary DC compared to craniotomy (respectively, 71 vs. 74%). The most frequently cited rationale for selecting a DC was a ‘pre-emptive approach to treatment of (suspected) raised ICP (not last resort)’ in 31% of DC cases (Appendix 18). The highest ICP on day 1 (day of surgery) and on day 2 of the patients with a primary DC was 12 (median, IQR 2–27) and 17 (median, IQR 11–24) respectively (Appendix 15–17). The proportion of DC patients that had a median day 1 ICP> 25 was 7/91 patients.

Secondary DC or craniotomy for contusions or hematomas was performed in 25 (27%) patients initially treated with primary DC and in 43 (18%) patients initially treated with craniotomy. Patients undergoing primary DC vs. craniotomy had longer hospital stays (median 38 vs. 18 days), more frequently required shunts (11 vs. 5%), and had more intracranial complications (delayed intracranial hematoma/seroma, 23 vs. 16%). Cranioplasty during primary admission was performed in 23 (25%) patients in the primary DC group and in 12 (5%) in the craniotomy group (after secondary DC) (Appendix 19–20).

**Fig. 2:**
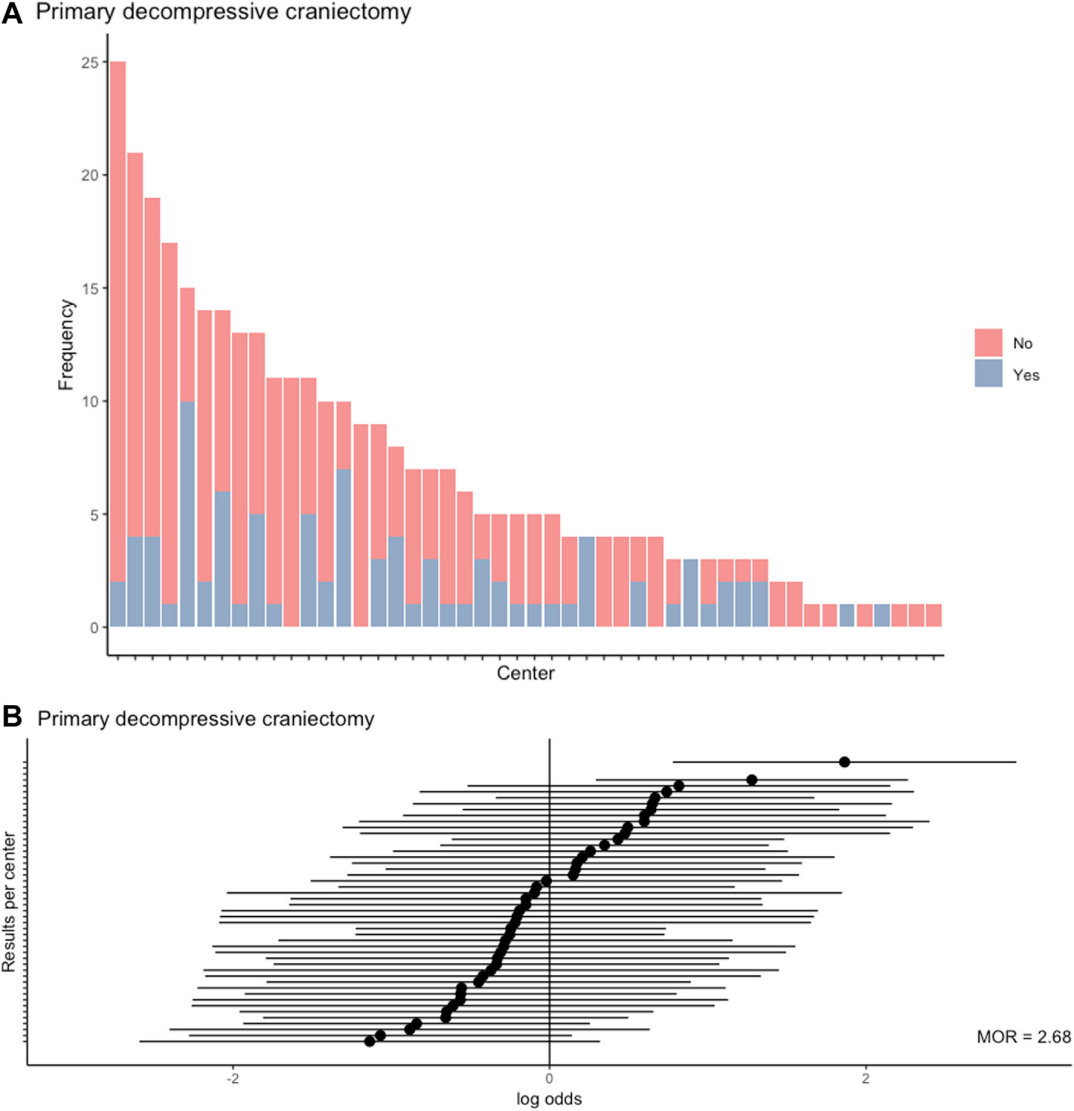

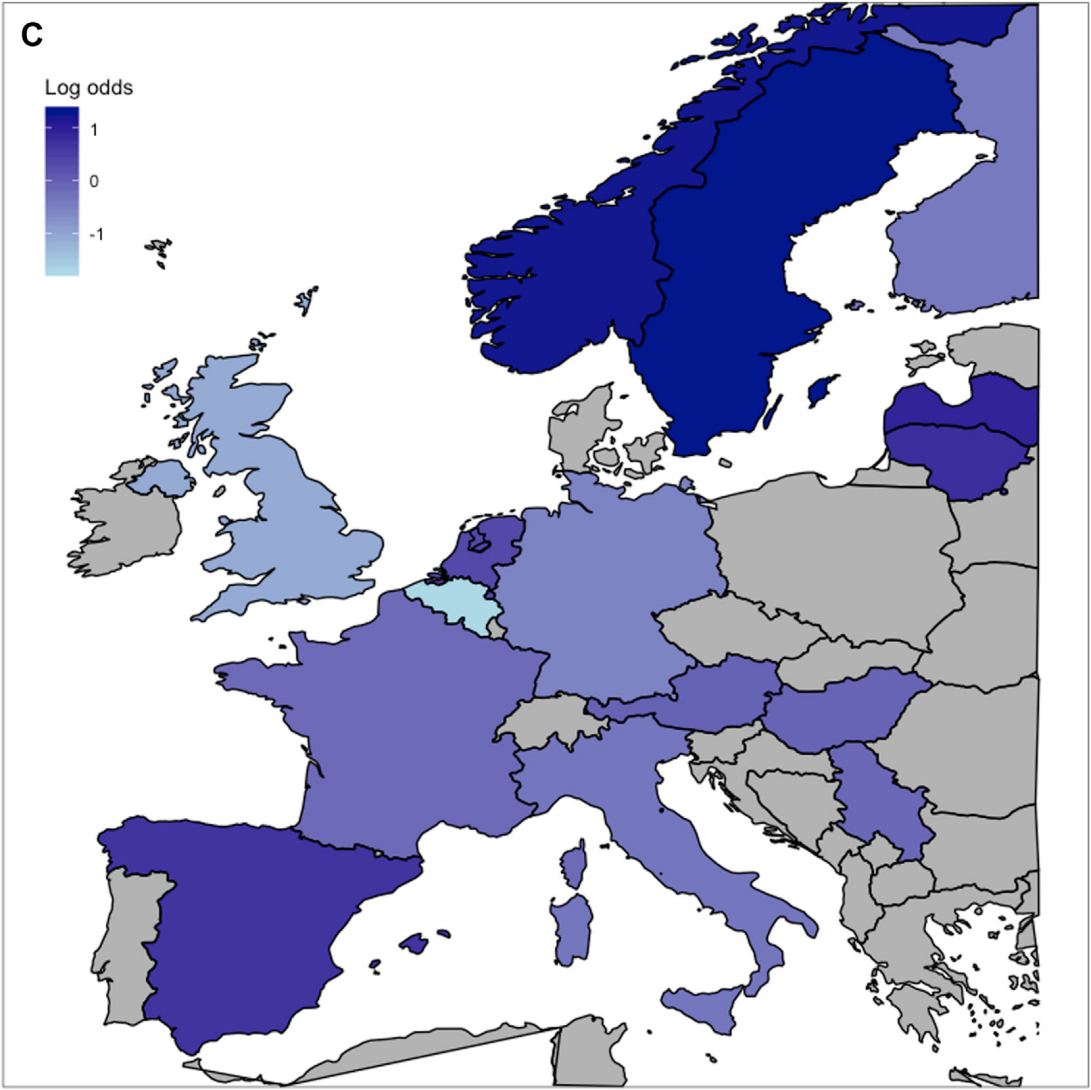
**Between-centre and between-country differences in primary decompressive craniectomy**. A) shows the observed frequencies of primary decompressive craniectomy per centre among patient who received surgical ASDH evacuation. B) shows the case-mix adjusted log odds ratio for primary decompressive craniectomy per centre. The median odds ratio (MOR) reflects the between-centre variation; a MOR equal to 1 represents no variation, the larger the MOR, the larger the variation. The MOR is 2.7 (*p* value < 0.0001). C) represents the log odds ratio for primary decompressive craniectomy as compared to craniotomy per country compared with the overall average, also case-mix adjusted.

The proportion of primary DC relative to all acute surgeries ranged from 6% to 67% across 46 centres (IQR = 12–26%; Fig. 2A), with a MOR of 2.7 (*p* < 0.0001) (Fig. 2B and C, Appendix 14 and 33), representing an almost 3-fold higher odds of receiving DC for clinically similar patients, when randomly comparing 2 centres. When ordering centres according to treatment preference, the baseline predicted 6-month functional outcome of the CRASH-CT score was similar across these centres despite differences in baseline characteristics (Table 1). The testable assumptions for IV analyses were met (Appendix 14 and 21).

**Table 1:** Selected baseline characteristics and prognosis across centers with different preferences for primary decompressive craniectomy.

	Treatment preference (observed primary DC rates per centre)^a^				
	Quartile 1 (6–12%)	Quartile 2 (12–19%)	Quartile 3 (19–26%)	Quartile 4 (26–67%)	SMD
n	53	48	51	48	
Age (median [IQR])	63 [56, 69]	56 [43, 66]	56 [38, 68]	53 [34, 64]	0.26
Sex					0.29
Female	12 (23)	15 (31)	23 (45)	9 (19)	
Male	41 (77)	33 (69)	28 (55)	39 (81)	
ASAPS (%)					0.44
Healthy	28 (53)	17 (35)	28 (55)	23 (48)	
Mild systemic disease	21 (40)	16 (33)	14 (27)	15 (31)	
Severe systemic disease	4 (8)	11 (23)	7 (14)	7 (15)	
Threat to life	0 (0)	0 (0)	1 (2)	0 (0)	
Unknown	0 (0)	4 (8)	1 (2)	3 (6)	
Hypoxia (%)^b^					0.49
No	41 (77)	42 (88)	44 (86)	37 (77)	
Definite	1 (2)	1 (2)	5 (10)	6 (12)	
Suspect	2 (4)	4 (8)	1 (2)	2 (4)	
Unknown	9 (17)	1 (2)	1 (2)	3 (6)	
Hypotension (%)^c^					0.44
No	41 (77)	46 (96)	46 (90)	39 (81)	
Definite	2 (4)	1 (2)	3 (6)	2 (4)	
Suspect	1 (2)	0 (0)	1 (2)	3 (6)	
Unknown	9 (17)	1 (2)	1 (2)	4 (8)	
Any major extracranial injury (%)^d^	23 (43)	15 (31)	28 (55)	25 (52)	0.27
GCS baseline (median [IQR])	9 [4, 13]	7 [3, 11]	5 [3, 9]	6 [3, 11]	0.30
GCS motor baseline (median [IQR])	5 [2, 6]	4 [1, 5]	1 [1, 4]	2 [1, 5]	0.43
Pupils (%)					0.32
Both reacting	36 (68)	35 (73)	32 (63)	28 (58)	
One reacting	3 (6)	7 (15)	9 (18)	10 (21)	
Both unreacting	14 (26)	6 (12)	10 (20)	10 (21)	
Any neuroworsening before surgery (%)	15 (28)	11 (23)	13 (25)	16 (33)	0.12
Total volume of ASDH (cm^3^, median [IQR])	58 [31, 97]	70 [40, 114]	70 [32, 103]	50 [18, 79]	0.24
CT large ASDH (%)^e^	35 (66)	37 (77)	42 (82)	31 (65)	0.25
CT midline shift (%)^f^	42 (79)	38 (79)	48 (94)	44 (92)	0.29
CT contusion (%)					0.40
No	19 (36)	25 (52)	21 (41)	23 (48)	
Small	24 (45)	19 (40)	23 (45)	13 (27)	
Large	10 (19)	3 (6)	6 (12)	11 (23)	
Unknown	0 (0)	1 (2)	1 (2)	1 (2)	
CT subarachnoid haemorrhage (%)					0.37
No	18 (34)	21 (44)	12 (24)	21 (44)	
Basal	5 (9)	2 (4)	6 (12)	2 (4)	
Cortical	22 (42)	15 (31)	27 (53)	16 (33)	
Basal and cortical	8 (15)	10 (21)	6 (12)	9 (19)	
CT basal cisterns absent/compressed (%)	20 (38)	19 (40)	25 (49)	21 (44)	0.13
Mean predicted 6-month unfavourable outcome (GOS score ≤3, %, median [IQR])^g^	74 [52, 86]	73 [53, 87]	80 [67, 91]	69 [51, 84]	0.22
Centre characteristics					
Academic hospital (vs. non- academic, %)	0 (0)	0 (0)	0 (0)	14 (29)	0.45
Number of beds (median [IQR])	655 [600, 850]	1083 [1018, 1148]	1170 [936, 1292]	780 [652, 831]	0.80
Residency program neurosurgery (%)	53 (100)	48 (100)	51 (100)	48 (100)	<0.01
Level I trauma centre designation (%)	42 (100)	48 (100)	51 (100)	35 (100)	<0.01
Urban location (vs. suburban and rural location, %)	53 (100)	48 (100)	51 (100)	48 (100)	<0.01
Neurosurgeon staffing (FTE, median [IQR])	11 [8, 19]	11 [10, 12]	10 [6, 12]	8 [8, 11]	0.50
Number of surgeries for ASDH in 2013 (median [IQR])	28 [10, 30]	18 [16, 20]	62 [20, 102]	25 [22, 25]	0.82
Number of surgeries for contusion in 2013 (median [IQR])	7 [5, 8]	10 [7, 14]	15 [4, 236]	10 [8, 14]	0.37
Low threshold policy for primary DC in ASDH (%)^h^	0 (0)	0 (0)	0 (0)	24 (50)	0.71

Abbreviation: AIS, Abbreviated Injury Scale; ASAPS, American Society of Anesthesiologists classification system; ASDH, acute subdural hematoma; DC, decompressive craniectomy; GCS, Glasgow Coma Scale; GOS, Glasgow Outcome Scale (5-point); IQR, interquartile range; IV, instrumental variable; SMD, standardised mean difference.

a Treatment preference as defined by the case-mix adjusted probability of undergoing primary DC (as opposed to craniotomy) based on the observed primary DC rates per centre. This corresponds to the IV status and presented in quartiles of the range of adjusted regional primary DC rates. The first category is less aggressive than the second and the second is less aggressive than the third and so forth. Importantly, the IV analysis used adjusted primary DC rates as continuous preference, the quartiles are presented for purposes of interpretability of baseline comparability.

b Second insult during the pre-hospital or ER phase, defined as PaO2 < 8 kPa (60 mmHg)/SaO2 < 90%. ‘Suspected’ was scored if the patient did not have documented hypoxia by PaO2 or SaO2, but there was a clinical suspicion, as evidenced by for example cyanosis, apnoea or respiratory distress.

c Second insult during the pre-hospital or ER phase, defined as systolic BP < 90 mmHg. ‘Suspected’ was scored if the patient did not have a documented blood pressure, but was reported to be in shock or have an absent brachial pulse (not related to injury of the extremity).

d AIS ≥3.

e Large is defined qualitatively by the treating neurosurgeon and corresponded to a size larger than 25 cm³.

f Midline shift present is classified as being more than 5 mm.

g TBI severity as summarised in predicted unfavorable outcome, proportion with a Glasgow Outcome Scale ≤3, based on CRASH-CT variables age, GCS score, pupillary reactivity to light, major extracranial injury, and CT characteristics (midline shift >5 mm, traumatic subarachnoid hemorrhage, and obliteration of the basal cisterns).

h Before patient inclusion in CENTER-TBI, treatment policies per centre were captured by provider profile surveys, including the policy towards primary DC. The resulting threshold for primary DC is dichotomised based on this distinction: Yes', primary DC routinely/pre-emptively versus ‘No’, no primary DC routinely/pre-emptively.

200 patients were available for primary IV analysis, after excluding patients from centres with <10 patients (n = 132). Centre preference for DC over craniotomy was not associated with better functional outcome (adjusted common OR 0.9, 95% CI 0.7–1.1 in favour of craniotomy, Table 2, Appendix 32). The aORs were consistent across GOSE cut-offs (Table 2). In-hospital mortality was also similar (aOR per 14% IQR more primary DC 1.3 [95% CI (1.0–3.4), Table 2). The association between surgical strategy and quality of life could not be estimated due to low numbers (only 1 centre with ≥10 patients in the QOLIBRI subgroup).

**Table 2:** Primary and secondary outcomes and treatment associations for primary decompressive craniectomy.

Outcome	Adjusted centre-level analyses					
	Treatment preference (observed primary DC rates per centre)				Effect variable	Adjusted value (95% CI)^a^
	Quartile 1 (6–12%, n = 53)	Quartile 2 (12–19%, n = 48)	Quartile 3 (19–26%, n = 51)	Quartile 4 (26–67%, n = 48)		
Primary outcome: GOSE at 6 months (median [IQR])	3 [1–7]	3 [1–6]	3 [1–6]	3 [1–6]	Common odds ratio	0.9 (0.7–1.1)
Secondary outcomes						
In-hospital mortality	12 (23)	11 (23)	21 (41)	18 (38)	Odds ratio	1.3 (1.0–3.4)
GOSE of 7 or 8 (%)	13 (25)	10 (21)	7 (14)	8 (17)	Odds ratio	0.9 (0.7–1.2)
GOSE of 5-8 (%)	17 (32)	20 (42)	16 (31)	17 (35)	Odds ratio	1.0 (0.8–1.2)
GOSE of 4-8 (%)	20 (38)	21 (44)	19 (37)	22 (46)	Odds ratio	1.0 (0.8–1.2)
QOLIBRI (median [IQR]) at 6 months^b^	Na^c^					

Abbreviation: CI, confidence interval; DC, decompressive craniectomy; GOSE, Glasgow Outcome Scale Extended; IQR, interquartile range; Na, not available; QOLIBRI, Quality of Life after Brain Injury Scale.

a Estimates from random-effect multivariable ordinal/logistic regression with the instrument, adjusted probability of undergoing primary DC as treatment variable. Confounding was furthermore addressed by adjusting for the a-priori defined variables age, GCS, pupil reactivity, hematoma size, contusion presence and midline shift. The adjusted common OR indicates the odds of a higher GOSE score (primary outcome) or experiencing the secondary outcomes, for an increase from the 25th percentile to the 75th percentile of the range in exposure to the centre intervention preferences.

b QOLIBRI is a standardised health specific quality of life measure specifically designed for and validated in outcome assesment in patients with brain injury. It is a numerical scale with scores ranging from 0 to 100, with higher scores indicating a better quality of life. The score was available for 19 patients of the primary DC group and 111 of the craniotomy group.

c The association could not be estimated due to low numbers (no centers with ≥10 patients in the subcohort).

After surgery, patients from centres that had a higher preference for DC more often had a period of neuroworsening and a higher Therapy Intensity Level (TIL). Otherwise, secondary outcomes did not differ between surgical preference groups (Appendix 22). The predominant reason for secondary DC after (primary) craniotomy was raised ICP (50%), while 50% of patients that needed a secondary DC after primary DC had a large ASDH (still or that reoccurred), 29% a large contusion and 29% had raised ICP as motivation for the DC. After secondary DC, three patients (12%) reached a GOSE >4 (Appendix 34).

Primary DC was associated with worse functional outcomes in unadjusted patient-level analysis (e.g., GOSE: common OR 0.4, 95% CI 0.3–0.6; Appendix 24 and 25). Covariable adjustment in multivariable regression and PSM (at patient-level) resulted in GOSE-association estimates favouring a craniotomy (adjusted common OR 0.4, 95% CI 0.2–0.6 and adjusted common OR 0.4, 95% CI 0.3–0.8, respectively; Appendix 23, 24, 26–27).

In sensitivity IV analyses, the primary association estimate remained consistent when excluding centres with <15 patients (n = 97; adjusted common OR 0.9 [95% CI 0.5–1.5], Appendix 24, 28–31), when using *a priori* defined IV (adjusted common OR 0.9, 95% CI 0.4–2.2; Appendix 24), and also in post-hoc analysis when excluding patients with poor baseline prognosis (adjusted common OR 0.9, 95% CI 0.7–1.1; Appendix 24). In-hospital mortality did not differ across centres with primary DC preference in sensitivity IV analysis excluding centres with <15 patients (Appendix 30). QOLIBRI scores were similar for both approaches, in unadjusted and adjusted (patient-level) analyses (Appendix 14).

The predominant reason for secondary DC after (primary) craniotomy was raised ICP (50%), while 50% of patients that needed a secondary DC after primary DC had a large ASDH (still or reoccurring), 29% a large contusion and 29% had raised ICP as motivation for the DC. After secondary DC, three patients (12%) reached a GOSE >4 (Appendix 34).

## Discussion

This prospective observational study demonstrates large treatment variation across European and Israeli centres in the selection of DC versus craniotomy in surgical evacuation of traumatic ASDH. Using this variation in IV analyses, we found that primary DC compared to craniotomy was unlikely to be associated with better functional outcome. These findings held in predefined IV sensitivity analyses, admittedly based on a small sample. Patient-level analysis with multivariable regression and PSM revealed poorer functional outcomes for primary DC, likely explained by residual confounding. Further, primary DC was associated with more complications and more follow-on surgeries.

Election of primary DC is well established in cases of ASDH with acutely severe swelling preventing replacement of the bone flap. A recent consensus states that if the brain is bulging beyond the inner table of the skull intra-operatively, the bone flap should not be replaced.^8^^,^^25^ The advantage of a DC is more effective control of ICP elevation, potentially preventing secondary brain injury and poor clinical outcome. However, DC necessitates additional reconstructive surgery (cranioplasty) and carries risks related to the bone defect, infections, and bone-flap reabsorption.^7^ Further, DC is known to alter cerebrospinal fluid flow dynamics, and cerebral blood flow dynamics, both of which improve with replacement of the bone flap.^26^, ^27^, ^28^, ^29^

This study ran parallel to the just published randomised clinical trial RESCUE-ASDH in which the conclusion was that primary DC and craniotomy lead to similar functional outcome and quality of life.^9^ Our effect estimates are in line with those from RESCUE-ASDH and extend outside the controlled setting of the RCT.

Obviously, we described the current practice patterns of neurosurgeons without the recent evidence provided by RESCUE-ASDH. Clinically relevant evidence was weak for primary DC in ASDH; large treatment variations exist,^12^^,^^30^^,^^31^ and support for claims of effectiveness are inconsistent. Most of these observational studies suggest worse outcomes for primary DC.^1^^,^^4^^,^^10^^,^^11^^,^^19^^,^^32^^,^^33^ When comparing the preoperative and baseline characteristic of DC versus craniotomy cohorts, all studies show that patients selected to undergo DC are more likely to have lower GCS, more concomitant hematomas and therefore, and have a poorer prognosis at baseline. Neurosurgeons are therefore more likely to select DC for the more severely impaired patients in anticipation of potential cerebral swelling that is difficult to manage medically. The higher number of patients with poor prognosis undergoing DC suggests strong confounding within these observational studies and that interpretation of worse outcomes resulting from primary DC, rather than from worse baseline status, may be incorrect.^5^^,^^14^ The methodologically best—albeit small–observational study evaluating this treatment variation through comparison of two neurosurgical centres found postoperative ICP to be better controlled and outcomes improved in the centres with greater utilisation of primary DC in TBI.^18^ However, these results included patients undergoing emergent DC or craniotomy for any mass or diffuse lesion, not specifically ASDH, which represented only 15 of 52 participants.

Our findings confirm these previously reported treatment variations and the inconsistency displayed by neurosurgeons as to selection of primary DC versus craniotomy in the absence of massive swelling. Patients in our cohort who underwent primary DC were also more severely injured, despite scoring similar prognoses on CRASH-CT, and required more interventions to lower ICP (i.e., greater TIL). Although many patients who received primary DC attained similar 6-month GOSE outcomes as patients who received craniotomy, as noted, they experienced a worse clinical course. Our study corroborates the findings of RESCUE-ASDH, provides real-world evidence that is generalisable to a broad population and shows that refraining from pre-emptive DC is safe in routine clinical practice.

Comparative effectiveness research with IV analysis, utilising heterogeneity in practices across centres to compare their effectiveness of interventions that may be standard practice in some centres, but not in others, offers complementary evidence to the gold standard of RCTs.^14^^,^^34^^,^^35^ Our similar results to RESCUE-ASDH attests to the strength of observational CER, although potential discrepancies could have also been the result of methodological choices and would necessarily mean persistent bias.^36^ Compared with conventional, patient-level analysis, IV CER is less prone to confounding. The validity, however, relies on whether the centre treatment rate is an appropriate instrumental variable. Our instrument was strongly associated with primary DC and did not associate with baseline prognosis: the widely differing surgical strategies are practiced in centres that on average treat similar patients. The balanced confounding between centres suggests a reasonable balance in the distribution of unmeasured confounding.^14^ Nonetheless, the observed practice variation might still partly result from prognostic differences. Therefore, we surveyed providers to evaluate whether the between-centre variation actually arose from provider preferences.^12^ The *a priori* reported centre policy for primary DC strongly predicted actual primary DC use (i.e., stronger than any single patient characteristic). To further extricate the effect of the ASDH surgical strategy in a centre from other between-centre care variations associated with outcome, we adjusted with a random-effects for centre.

Generalisability of our findings requires careful consideration. First, estimating an overall effect of any (surgical) intervention in TBI is amenable to a neutral result due to averaging heterogeneous effects.^37^ In acute neurosurgery several RCTs and comparative observational studies have reported neutral findings.^9^^,^^33^^,^^38^^,^^39^

Second, our IV analysis applies to patients for whom the neurosurgeon may be in equipoise, judging that more than one valid treatment option exists. As this equipoise differs per centre, we cannot readily identify the relative contribution of each subgroup.^40^ IV analysis therefore, may predominantly provide information on whether patients' outcome will improve when centres change their policy with respect to a specific intervention. Estimating an effect in individual patients is difficult, especially in light of our different patient-level results that suggest worse outcomes.^41^^,^^42^ Thus, although the inherent heterogeneity in ASDH and the indefinable patient population in IV effect estimation precludes recognising an individual treatment effect, the results suggest, when in equipoise regarding the decision to leave the bone flap on or off, no difference in outcome due to a centre's treatment strategy. Given the higher risk of a complicated clinical course, the selection of primary DC should be restricted to salvageable patients with brain swelling precluding flap repositioning.

We acknowledge several other limitations. First, possible residual confounding remains due to other local practice variations associated with surgical preference, despite IV analysis, rigorous statistical adjustment (i.e., a random-effects term) and multiple sensitivity analyses, particularly the IV analysis with the *a priori* centre policy for approach as a different, strong IV, strongly correlated to the actual DC employment, confirming consistent neurosurgeon's preferences. To further account for centre-level confounding we performed a separate cluster analysis, with a broader medical domain view than neurosurgical treatment alone, to explore if the assumption of the absence of correlation between treatment choices is tenable. The main conclusion was that specific treatment policies within domains (ICP monitoring, coagulation and transfusion, neurosurgery, prophylactic antibiotics, and more general ICU treatment policies) do not correlate with other treatment policies. Importantly, the absence of correlation between domains was most pronounced for surgical treatment. Another limitation is that participating institutions of CENTER-TBI were mainly tertiary referral centres. Results may not be generalisable to other hospital settings and every patient. A limitation is also the missing data on the size of the craniectomy, which hampered informative analysis. Last, the interpretation of the effect of primary DC is hampered by the relatively small sample size, resulting in a wide confidence interval that may obscure a small, clinically relevant effect. Although this is the largest observational cohort to date, subgroup analyses were considered infeasible.

Large treatment variation exists across European and Israeli centres in the selection of DC versus craniotomy in surgical evacuation of traumatic ASDH. Comparing the differing strategies, surgical evacuation by primary DC as compared to craniotomy may unlikely be of benefit, measured in terms of functional outcome at 6 months, and has a higher risk of complications. Our study underscores the necessity of collecting granular data on interventions and their sequelae to more accurately delineate the clinical course as a follow up to the recently published RCT RESCUE-ASDH trial. Guidelines might restrict primary DC to patients whose intraoperative brain swelling precludes replacement of the bone flap for more consistency in current practice.

## Contributors

TAvE conceptualised the study, curated the data, analysed the data, and drafted the manuscript including all tables and figures. IAMvE assisted in the analysis and draft of the manuscript. IAMvE and DP assisted in the data curation. RDS, JTJMD, JKY, AK, GCWdR, HFL, PJAH, EWS, AIRM, and WCP assisted in the interpretation of the data and helped drafting the manuscript. AIRM, GCWdR, WP, HFL and EWS supervised the methodology of the study protocol and supervised the study. TAvE, HFL, VV, HFdB, JKY, AK, DKM, PJAH, BD, EWS, AIRM, GCWdR, and WCP were involved in the design of CENTER-TBI. All authors reviewed and approved the final version of the manuscript. TAvE, IAMvE, and DP accessed and verified the analyses. All authors guarantee that the manuscript is an honest, accurate, and transparent account of the study being reported and that no important aspects of the study have been omitted. All authors had full access to all the data in the study and all authors had final responsibility for the decision to submit for publication.

## Data sharing statement

The datasets, which include individual participant data and a data dictionary defining each field in the set used or analysed during the current study, will be available upon reasonable request to the management committee of the CENTER-TBI study. Requests for data should be submitted online at https://www.center-tbi.eu/data or via email to center-tbi@uza.be. The data that will be made available comprise deidentified participant data. The predefined study protocol is published. The statistical analysis plan, R syntax, and informed consent forms will be made available upon request. To access any other data from CENTER-TBI, a proposal should be submitted and approved by the management committees of the CENTER-TBI study. A data access agreement with the management team of CENTER-TBI should be signed before access to the data will be granted.

## Editor note

The Lancet Group takes a neutral position with respect to territorial claims in published maps and institutional affiliations.

## Declaration of interests

AIRM declares consulting fees from PresSura Neuro, Integra Life Sciences, and NeuroTrauma Sciences. DKM reports grants from the UK National Institute for Health Research, during the conduct of the study; grants, personal fees, and non-financial support from GlaxoSmithKline; and personal fees from Neurotrauma Sciences, Lantmaanen AB, Pressura, and Pfizer, outside of the submitted work. EWS reports personal fees from Springer during the conduct of the study. VV reports JAMA Statistical Reviewer Board membership. All other authors declare no competing interests.

## Appendix A Supplementary data

Supplementary Appendix

CENTER-TBI group members

## Footnotes

o The CENTER-TBI patients enrolled in The Netherlands were co-enrolled in the Neurotraumatology Quality Registry (Net-QuRe), which enrolled patients between 2015 and 2020 in 7 centers across the Netherlands. Net-QuRe had identical eligibility criteria but included patients with a Glasgow Coma Scale (GCS) score <13. Net-QuRe is registered on the International Clinical Trial Registry Platform (NTR6003) and the Dutch Trial Register (NL5761).
