# Supplementary material for: Comparative effectiveness of decompressive craniectomy versus craniotomy for traumatic acute subdural hematoma (CENTER-TBI): an observational cohort study
Source: eClinicalMedicine. 2023 Aug 9;63:102161. doi: 10.1016/j.eclinm.2023.102161 (PMC10432786; doi:10.1016/j.eclinm.2023.102161)
Supplement: Supplementary Appendix [file mmc1.docx]

**Supplementary appendix**

**The CENTER-TBI participants and investigators (page 2-11)**

**Additional methods (page 12-13)**

**Additional results (page 14)**

**Supplemental tables and figures (page 15-35):**

**Supplemental Table 1. Baseline and treatment characteristics of patients with acute surgery for traumatic acute subdural hematoma, comparing primary decompressive craniectomy and craniotomy**

**Supplemental Table 2. Characteristics of primary decompressive craniectomy**

**Supplemental Table 3. Hospital course, complications and follow-up of patients with acute surgery for traumatic acute subdural hematoma, comparing primary decompressive craniectomy and craniotomy**

**Supplemental Table 4. Assumptions for instrumental variable analyses**

**Supplemental Table 5. Hospital course and outcome across centers for primary decompressive craniectomy**

**Supplemental Table 6. Results of sensitivity analyses: comparing analytical methods to adjust for confounding by indication in proportional odds logistic regression model with the Glasgow Outcome Scale Extended score as outcome**

**Supplemental Figure 1. Propensity scores distribution of nonmatched cohorts and propensity matched cohorts of primary decompressive craniectomy**

**Supplemental Table 7. Unadjusted patient-level analysis**

**Supplemental Table 8: Baseline characteristics of propensity matched cohort, comparing primary decompressive craniectomy with craniotomy**

**Supplemental Table 9. Selected baseline characteristics and prognosis across centers for primary decompressive craniectomy, excluding centers < 15 patients**

**Supplemental Table 10. Hospital course and outcome across centers for primary decompressive craniectomy, excluding centers < 15 patients**

**Supplemental Table 11. Primary and secondary outcomes and treatment associations for primary decompressive craniectomy, excluding centers < 15 patients**

**Supplemental Figure 2. Functional outcome with different probabilities of primary decompressive craniectomy.**

**Supplemental Figure 3. Between-centre differences in primary decompressive craniectomy excluding outlying centres.**

**Supplemental Table 12. CT characteristics, surgical motivations and outcome of patients who received secondary decompressive craniectomy**

**References (page 36)**

**The CENTER-TBI participants and investigators**

Cecilia Åkerlund^1^, Krisztina Amrein^2^, Nada Andelic^3^, Lasse Andreassen^4^, Audny Anke^5^, Anna Antoni^6^, Gérard Audibert^7^, Philippe Azouvi^8^, Maria Luisa Azzolini^9^, Ronald Bartels^10^, Pál Barzó^11^, Romuald Beauvais^12^, Ronny Beer^13^, Bo-Michael Bellander^14^, Antonio Belli^15^, Habib Benali^16^, Maurizio Berardino^17^, Luigi Beretta^9^, Morten Blaabjerg^18^, Peter Bragge^19^, Alexandra Brazinova^20^, Vibeke Brinck^21^, Joanne Brooker^22^, Camilla Brorsson^23^, Andras Buki^24^, Monika Bullinger^25^, Manuel Cabeleira^26^, Alessio Caccioppola^27^, Emiliana Calappi ^27^, Maria Rosa Calvi^9^, Peter Cameron^28^, Guillermo Carbayo Lozano^29^, Marco Carbonara^27^, Simona Cavallo^17^, Giorgio Chevallard^30^, Arturo Chieregato^30^, Giuseppe Citerio^31, 32^, Hans Clusmann^33^, Mark Coburn^34^, Jonathan Coles^35^, Jamie D. Cooper^36^, Marta Correia^37^, Amra Čović ^38^, Nicola Curry^39^, Endre Czeiter^24^, Marek Czosnyka^26^, Claire Dahyot‑Fizelier^40^, Paul Dark^41^, Helen Dawes^42^, Véronique De Keyser^43^, Vincent Degos^16^, Francesco Della Corte^44^, Hugo den Boogert^10^, Bart Depreitere^45^, Đula Đilvesi ^46^, Abhishek Dixit^47^, Emma Donoghue^22^, Jens Dreier^48^, Guy‑Loup  Dulière^49^, Ari Ercole^47^, Patrick Esser^42^, Erzsébet Ezer^50^, Martin  Fabricius^51^, Valery L. Feigin^52^, Kelly  Foks^53^, Shirin Frisvold^54^, Alex Furmanov^55^, Pablo Gagliardo^56^, Damien Galanaud^16^, Dashiell Gantner^28^, Guoyi Gao^57^, Pradeep George^58^, Alexandre Ghuysen^59^, Lelde Giga^60^, Ben Glocker^61^, Jagoš Golubovic^46^, Pedro A. Gomez ^62^, Johannes Gratz^63^, Benjamin Gravesteijn^64^, Francesca Grossi^44^, Russell L. Gruen^65^, Deepak Gupta^66^, Juanita A. Haagsma^64^, Iain Haitsma^67^, Raimund Helbok^13^, Eirik Helseth^68^, Lindsay Horton ^69^, Jilske Huijben^64^, Peter J. Hutchinson^70^, Bram Jacobs^71^, Stefan Jankowski^72^, Mike Jarrett^21^, Ji‑yao  Jiang^58^, Faye Johnson^73^, Kelly Jones^52^, Mladen Karan^46^, Angelos G. Kolias^70^, Erwin Kompanje^74^, Daniel Kondziella^51^, Evgenios Kornaropoulos^47^, Lars‑Owe Koskinen^75^, Noémi Kovács^76^, Ana Kowark^77^, Alfonso Lagares^62^, Linda Lanyon^58^, Steven Laureys^78^, Fiona Lecky^79, 80^, Didier Ledoux^78^, Rolf Lefering^81^, Valerie Legrand^82^, Aurelie Lejeune^83^, Leon Levi^84^, Roger Lightfoot^85^, Hester Lingsma^64^, Andrew I.R. Maas^43^, Ana M. Castaño‑León^62^, Marc Maegele^86^, Marek Majdan^20^, Alex Manara^87^, Geoffrey Manley^88^, Costanza Martino^89^, Hugues Maréchal^49^, Julia Mattern^90^, Catherine McMahon^91^, Béla Melegh^92^, David Menon^47^, Tomas Menovsky^43^, Ana Mikolic^64^, Benoit Misset^78^, Visakh Muraleedharan^58^, Lynnette Murray^28^, Ancuta Negru^93^, David Nelson^1^, Virginia Newcombe^47^, Daan Nieboer^64^, József Nyirádi^2^, Otesile Olubukola^79^, Matej Oresic^94^, Fabrizio Ortolano^27^, Aarno Palotie^95, 96, 97^, Paul M. Parizel^98^, Jean‑François Payen^99^, Natascha Perera^12^, Vincent Perlbarg^16^, Paolo Persona^100^, Wilco Peul^101^, Anna Piippo-Karjalainen^102^, Matti Pirinen^95^, Dana Pisica^64^, Horia Ples^93^, Suzanne Polinder^64^, Inigo Pomposo^29^, Jussi P. Posti ^103^, Louis Puybasset^104^, Andreea Radoi ^105^, Arminas Ragauskas^106^, Rahul Raj^102^, Malinka Rambadagalla^107^, Isabel Retel Helmrich^64^, Jonathan Rhodes^108^, Sylvia Richardson^109^, Sophie Richter^47^, Samuli Ripatti^95^, Saulius Rocka^106^, Cecilie Roe^110^, Olav Roise^111,112^, Jonathan Rosand^113^, Jeffrey V. Rosenfeld^114^, Christina Rosenlund^115^, Guy Rosenthal^55^, Rolf Rossaint^77^, Sandra Rossi^100^, Daniel Rueckert^61^ Martin Rusnák^116^, Juan Sahuquillo^105^, Oliver Sakowitz^90, 117^, Renan Sanchez‑Porras^117^, Janos Sandor^118^, Nadine Schäfer^81^, Silke Schmidt^119^, Herbert Schoechl^120^, Guus Schoonman^121^, Rico Frederik Schou^122^, Elisabeth Schwendenwein^6^, Charlie Sewalt^64^, Toril Skandsen^123, 124^ , Peter Smielewski^26^, Abayomi Sorinola^125^, Emmanuel Stamatakis^47^, Simon Stanworth^39^, Robert Stevens^126^, William Stewart^127^, Ewout W. Steyerberg^128^, Nino Stocchetti^129^, Nina Sundström^130^, Riikka Takala^131^, Viktória Tamás^125^, Tomas Tamosuitis^132^, Mark Steven Taylor^20^, Braden Te Ao^52^, Olli Tenovuo^103^, Alice Theadom^52^, Matt Thomas^87^, Dick Tibboel^133^, Marjolein Timmers^74^, Christos Tolias^134^, Tony Trapani^28^, Cristina Maria Tudora^93^, Andreas Unterberg^90^, Peter Vajkoczy ^135^, Shirley Vallance^28^, Egils Valeinis^60^, Zoltán Vámos^50^, Mathieu van der Jagt^136^, Gregory Van der Steen^43^, Joukje van der Naalt^71^, Jeroen T.J.M. van Dijck ^101^, Thomas A. van Essen^101^, Wim Van Hecke^137^, Caroline van Heugten^138^, Dominique Van Praag^139^, Ernest van Veen^64^, Thijs Vande Vyvere^137^, Roel P. J. van Wijk^101^, Alessia Vargiolu^32^, Emmanuel Vega^83^, Kimberley Velt^64^, Jan Verheyden^137^, Paul M. Vespa^140^, Anne Vik^123, 141^, Rimantas Vilcinis^132^, Victor Volovici^67^, Nicole von Steinbüchel^38^, Daphne Voormolen^64^, Petar Vulekovic^46^, Kevin K.W. Wang^142^, Eveline Wiegers^64^, Guy Williams^47^, Lindsay Wilson^69^, Stefan Winzeck^47^, Stefan Wolf^143^, Zhihui Yang^113^, Peter Ylén^144^, Alexander Younsi^90^, Frederick A. Zeiler^47,145^, Veronika Zelinkova^20^, Agate Ziverte^60^ , Tommaso Zoerle^27^

^1^ Department of Physiology and Pharmacology, Section of Perioperative Medicine and Intensive Care, Karolinska Institutet, Stockholm, Sweden

^2^ János Szentágothai Research Centre, University of Pécs, Pécs, Hungary

^3^ Division of Surgery and Clinical Neuroscience, Department of Physical Medicine and Rehabilitation, Oslo University Hospital and University of Oslo, Oslo, Norway

^4^ Department of Neurosurgery, University Hospital Northern Norway, Tromso, Norway

^5^ Department of Physical Medicine and Rehabilitation, University Hospital Northern Norway, Tromso, Norway

^6^ Trauma Surgery, Medical University Vienna, Vienna, Austria

^7^ Department of Anesthesiology & Intensive Care, University Hospital Nancy, Nancy, France

^8^ Raymond Poincare hospital, Assistance Publique – Hopitaux de Paris, Paris, France

^9^ Department of Anesthesiology & Intensive Care, S Raffaele University Hospital, Milan, Italy

^10^ Department of Neurosurgery, Radboud University Medical Centre, Nijmegen, The Netherlands

^11^ Department of Neurosurgery, University of Szeged, Szeged, Hungary

^12^ International Projects Management, ARTTIC, Munchen, Germany

^13^ Department of Neurology, Neurological Intensive Care Unit, Medical University of Innsbruck, Innsbruck, Austria

^14^ Department of Neurosurgery & Anesthesia & intensive care medicine, Karolinska University Hospital, Stockholm, Sweden

^15^ NIHR Surgical Reconstruction and Microbiology Research Centre, Birmingham, UK

^16^ Anesthesie-Réanimation, Assistance Publique – Hopitaux de Paris, Paris, France

^17^ Department of Anesthesia & ICU, AOU Città della Salute e della Scienza di Torino - Orthopedic and Trauma Center, Torino, Italy

^18^ Department of Neurology, Odense University Hospital, Odense, Denmark

^19^ BehaviourWorks Australia, Monash Sustainability Institute, Monash University, Victoria, Australia

^20^ Department of Public Health, Faculty of Health Sciences and Social Work, Trnava University, Trnava, Slovakia

^21^ Quesgen Systems Inc., Burlingame, California, USA

^22^ Australian & New Zealand Intensive Care Research Centre, Department of Epidemiology and Preventive Medicine, School of Public Health and Preventive Medicine, Monash University, Melbourne, Australia

^23^ Department of Surgery and Perioperative Science, Umeå University, Umeå, Sweden

^24^ Department of Neurosurgery, Medical School, University of Pécs, Hungary and Neurotrauma Research Group, János Szentágothai Research Centre, University of Pécs, Hungary

^25^ Department of Medical Psychology, Universitätsklinikum Hamburg-Eppendorf, Hamburg, Germany

^26^ Brain Physics Lab, Division of Neurosurgery, Dept of Clinical Neurosciences, University of Cambridge, Addenbrooke’s Hospital, Cambridge, UK

^27^ Neuro ICU, Fondazione IRCCS Cà Granda Ospedale Maggiore Policlinico, Milan, Italy

^28^ ANZIC Research Centre, Monash University, Department of Epidemiology and Preventive Medicine, Melbourne, Victoria, Australia

^29^ Department of Neurosurgery, Hospital of Cruces, Bilbao, Spain

^30^ NeuroIntensive Care, Niguarda Hospital, Milan, Italy

^31^ School of Medicine and Surgery, Università Milano Bicocca, Milano, Italy

^32^ NeuroIntensive Care, ASST di Monza, Monza, Italy

^33^Department of Neurosurgery, Medical Faculty RWTH Aachen University, Aachen, Germany

^34^ Department of Anesthesiology and Intensive Care Medicine, University Hospital Bonn, Bonn, Germany

^35^ Department of Anesthesia & Neurointensive Care, Cambridge University Hospital NHS Foundation Trust, Cambridge, UK

^36^ School of Public Health & PM, Monash University and The Alfred Hospital, Melbourne, Victoria, Australia

^37^ Radiology/MRI department, MRC Cognition and Brain Sciences Unit, Cambridge, UK

^38^ Institute of Medical Psychology and Medical Sociology, Universitätsmedizin Göttingen, Göttingen, Germany

^39^ Oxford University Hospitals NHS Trust, Oxford, UK

^40^ Intensive Care Unit, CHU Poitiers, Potiers, France

^41^ University of Manchester NIHR Biomedical Research Centre, Critical Care Directorate,  Salford Royal Hospital NHS Foundation Trust, Salford, UK

^42^ Movement Science Group, Faculty of Health and Life Sciences, Oxford Brookes University, Oxford, UK

^43^ Department of Neurosurgery, Antwerp University Hospital and University of Antwerp, Edegem, Belgium

^44^ Department of Anesthesia & Intensive Care, Maggiore Della Carità Hospital, Novara, Italy

^45^ Department of Neurosurgery, University Hospitals Leuven, Leuven, Belgium

^46^ Department of Neurosurgery, Clinical centre of Vojvodina, Faculty of Medicine, University of Novi Sad, Novi Sad, Serbia

^47^ Division of Anaesthesia, University of Cambridge, Addenbrooke’s Hospital, Cambridge, UK

^48^ Center for Stroke Research Berlin, Charité – Universitätsmedizin Berlin, corporate member of Freie Universität Berlin, Humboldt-Universität zu Berlin, and Berlin Institute of Health, Berlin, Germany

^49^ Intensive Care Unit, CHR Citadelle, Liège, Belgium

^50^ Department of Anaesthesiology and Intensive Therapy, University of Pécs, Pécs, Hungary

^51^ Departments of Neurology, Clinical Neurophysiology and Neuroanesthesiology, Region Hovedstaden Rigshospitalet, Copenhagen, Denmark

^52^ National Institute for Stroke and Applied Neurosciences, Faculty of Health and Environmental Studies, Auckland University of Technology, Auckland, New Zealand

^53^ Department of Neurology, Erasmus MC, Rotterdam, the Netherlands

^54^ Department of Anesthesiology and Intensive care, University Hospital Northern Norway, Tromso, Norway

^55^ Department of Neurosurgery, Hadassah-hebrew University Medical center, Jerusalem, Israel

^56^ Fundación Instituto Valenciano de Neurorrehabilitación (FIVAN), Valencia, Spain

^57^ Department of Neurosurgery, Shanghai Renji hospital, Shanghai Jiaotong University/school of medicine, Shanghai, China

^58^ Karolinska Institutet, INCF International Neuroinformatics Coordinating Facility, Stockholm, Sweden

^59^ Emergency Department, CHU, Liège, Belgium

^60^ Neurosurgery clinic, Pauls Stradins Clinical University Hospital, Riga, Latvia

^61^ Department of Computing, Imperial College London, London, UK

^62^ Department of Neurosurgery, Hospital Universitario 12 de Octubre, Madrid, Spain

^63^ Department of Anesthesia, Critical Care and Pain Medicine, Medical University of Vienna, Austria

^64^ Department of Public Health, Erasmus Medical Centre-University Medical Centre, Rotterdam, The Netherlands

^65^ College of Health and Medicine, Australian National University, Canberra, Australia

^66^ Department of Neurosurgery, Neurosciences Centre & JPN Apex trauma centre, All India Institute of Medical Sciences, New Delhi-110029, India

^67^ Department of Neurosurgery, Erasmus MC, Rotterdam, the Netherlands

^68^ Department of Neurosurgery, Oslo University Hospital, Oslo, Norway

^69^ Division of Psychology, University of Stirling, Stirling, UK

^70^ Division of Neurosurgery, Department of Clinical Neurosciences, Addenbrooke’s Hospital & University of Cambridge, Cambridge, UK

^71^ Department of Neurology, University of Groningen, University Medical Centre Groningen, Groningen, Netherlands

^72^ Neurointensive Care , Sheffield Teaching Hospitals NHS Foundation Trust, Sheffield, UK

^73^ Salford Royal Hospital NHS Foundation Trust Acute Research Delivery Team, Salford, UK

^74^ Department of Intensive Care and Department of Ethics and Philosophy of Medicine, Erasmus Medical Centre, Rotterdam, The Netherlands

^75^ Department of Clinical Neuroscience, Neurosurgery, Umeå University, Umeå, Sweden

^76^ Hungarian Brain Research Program - Grant No. KTIA_13_NAP-A-II/8, University of Pécs, Pécs, Hungary

^77^ Department of Anaesthesiology, University Hospital of Aachen, Aachen, Germany

^78^ Cyclotron Research Center , University of Liège, Liège, Belgium

^79^ Centre for Urgent and Emergency Care Research (CURE), Health Services Research Section, School of Health and Related Research (ScHARR), University of Sheffield, Sheffield, UK

^80^ Emergency Department, Salford Royal Hospital, Salford UK

^81^ Institute of Research in Operative Medicine (IFOM), Witten/Herdecke University, Cologne, Germany

^82^ VP Global Project Management CNS, ICON, Paris, France

^83^ Department of Anesthesiology-Intensive Care, Lille University Hospital, Lille, France

^84^ Department of Neurosurgery, Rambam Medical Center, Haifa, Israel

^85^ Department of Anesthesiology & Intensive Care, University Hospitals Southhampton NHS Trust, Southhampton, UK

^86^ Cologne-Merheim Medical Center (CMMC), Department of Traumatology, Orthopedic Surgery and Sportmedicine, Witten/Herdecke University, Cologne, Germany

^87^ Intensive Care Unit, Southmead Hospital, Bristol, Bristol, UK

^88^ Department of Neurological Surgery, University of California, San Francisco, California, USA

^89^ Department of Anesthesia & Intensive Care,M. Bufalini Hospital, Cesena, Italy

^90^ Department of Neurosurgery, University Hospital Heidelberg, Heidelberg, Germany

^91^ Department of Neurosurgery, The Walton centre NHS Foundation Trust, Liverpool, UK

^92^ Department of Medical Genetics, University of Pécs, Pécs, Hungary

^93^ Department of Neurosurgery, Emergency County Hospital Timisoara , Timisoara, Romania

^94^ School of Medical Sciences, Örebro University, Örebro, Sweden

^95^ Institute for Molecular Medicine Finland, University of Helsinki, Helsinki, Finland

^96^ Analytic and Translational Genetics Unit, Department of Medicine; Psychiatric & Neurodevelopmental Genetics Unit, Department of Psychiatry; Department of Neurology, Massachusetts General Hospital, Boston, MA, USA

^97^ Program in Medical and Population Genetics; The Stanley Center for Psychiatric Research, The Broad Institute of MIT and Harvard, Cambridge, MA, USA

^98^ Department of Radiology, University of Antwerp, Edegem, Belgium

^99^ Department of Anesthesiology & Intensive Care, University Hospital of Grenoble, Grenoble, France

^100^ Department of Anesthesia & Intensive Care, Azienda Ospedaliera Università di Padova, Padova, Italy

^101^ Dept. of Neurosurgery, Leiden University Medical Centre, Leiden, The Netherlands and Dept. of Neurosurgery, Medical Centre Haaglanden, The Hague, The Netherlands

^102^ Department of Neurosurgery, Helsinki University Central Hospital

^103^ Division of Clinical Neurosciences, Department of Neurosurgery and Turku Brain Injury Centre, Turku University Hospital and University of Turku, Turku, Finland

^104^ Department of Anesthesiology and Critical Care, Pitié -Salpêtrière Teaching Hospital, Assistance Publique, Hôpitaux de Paris and University Pierre et Marie Curie, Paris, France

^105^ Neurotraumatology and Neurosurgery Research Unit (UNINN), Vall d'Hebron Research Institute, Barcelona, Spain

^106^ Department of Neurosurgery, Kaunas University of technology and Vilnius University, Vilnius, Lithuania

^107^ Department of Neurosurgery, Rezekne Hospital, Latvia

^108^ Department of Anaesthesia, Critical Care & Pain Medicine NHS Lothian & University of Edinburg, Edinburgh, UK

^109^ Director, MRC Biostatistics Unit, Cambridge Institute of Public Health, Cambridge, UK

^110^ Department of Physical Medicine and Rehabilitation, Oslo University Hospital/University of Oslo, Oslo, Norway

^111^ Division of Orthopedics, Oslo University Hospital, Oslo, Norway

^112^ Institue of Clinical Medicine, Faculty of Medicine, University of Oslo, Oslo, Norway

^113^ Broad Institute, Cambridge MA Harvard Medical School, Boston MA, Massachusetts General Hospital, Boston MA, USA

^114^ National Trauma Research Institute, The Alfred Hospital, Monash University, Melbourne, Victoria, Australia

^115^ Department of Neurosurgery, Odense University Hospital, Odense, Denmark

^116^ International Neurotrauma Research Organisation, Vienna, Austria

^117^ Klinik für Neurochirurgie, Klinikum Ludwigsburg, Ludwigsburg, Germany

^118^ Division of Biostatistics and Epidemiology, Department of Preventive Medicine, University of Debrecen, Debrecen, Hungary

^119^ Department Health and Prevention, University Greifswald, Greifswald, Germany

^120^ Department of Anaesthesiology and Intensive Care, AUVA Trauma Hospital, Salzburg, Austria

^121^ Department of Neurology, Elisabeth-TweeSteden Ziekenhuis, Tilburg, the Netherlands

^122^ Department of Neuroanesthesia and Neurointensive Care, Odense University Hospital, Odense, Denmark

^123^ Department of Neuromedicine and Movement Science, Norwegian University of Science and Technology, NTNU, Trondheim, Norway

^124^ Department of Physical Medicine and Rehabilitation, St.Olavs Hospital, Trondheim University Hospital, Trondheim, Norway

^125^ Department of Neurosurgery, University of Pécs, Pécs, Hungary

^126^ Division of Neuroscience Critical Care, John Hopkins University School of Medicine, Baltimore, USA

^127^ Department of Neuropathology, Queen Elizabeth University Hospital and University of Glasgow, Glasgow, UK

^128^ Dept. of Department of Biomedical Data Sciences, Leiden University Medical Centre, Leiden, The Netherlands

^129^ Department of Pathophysiology and Transplantation, Milan University, and Neuroscience ICU, Fondazione IRCCS Cà Granda Ospedale Maggiore Policlinico, Milano, Italy

^130^ Department of Radiation Sciences, Biomedical Engineering, Umeå University, Umeå, Sweden

^131^ Perioperative Services, Intensive Care Medicine and Pain Management, Turku University Hospital and University of Turku, Turku, Finland

^132^ Department of Neurosurgery, Kaunas University of Health Sciences, Kaunas, Lithuania

^133^ Intensive Care and Department of Pediatric Surgery, Erasmus Medical Centre, Sophia Children’s Hospital, Rotterdam, The Netherlands

^134^ Department of Neurosurgery, Kings college London, London, UK

^135^ Neurologie, Neurochirurgie und Psychiatrie, Charité – Universitätsmedizin Berlin, Berlin, Germany

^136^ Department of Intensive Care Adults, Erasmus MC– University Medical Centre Rotterdam, Rotterdam, the Netherlands

^137^ icoMetrix NV, Leuven, Belgium

^138^ Movement Science Group, Faculty of Health and Life Sciences, Oxford Brookes University, Oxford, UK

^139^ Psychology Department, Antwerp University Hospital, Edegem, Belgium

^140^ Director of Neurocritical Care, University of California, Los Angeles, USA

^141^ Department of Neurosurgery, St.Olavs Hospital, Trondheim University Hospital, Trondheim, Norway

^142^ Department of Emergency Medicine, University of Florida, Gainesville, Florida, USA

^143^ Department of Neurosurgery, Charité – Universitätsmedizin Berlin, corporate member of Freie Universität Berlin, Humboldt-Universität zu Berlin, and Berlin Institute of Health, Berlin, Germany

^144^ VTT Technical Research Centre, Tampere, Finland

^145^ Section of Neurosurgery, Department of Surgery, Rady Faculty of Health Sciences, University of Manitoba, Winnipeg, MB, Canada

| Åkerlund | Cecilia | cecilia.ai.akerlund@gmail.com |
| --- | --- | --- |
| Amrein | Krisztina | tina.amrein84@gmail.com |
| Andelic | Nada | NADAND@ous-hf.no |
| Andreassen | Lasse | Lasse.Andreassen@unn.no |
| Anke | Audny | [Audny.anke@unn.no](mailto:Audny.anke@unn.no) |
| Antoni | Anna | [anna.antoni@meduniwien.ac.at](mailto:anna.antoni@meduniwien.ac.at) |
| Audibert | Gérard | g.audibert@chu-nancy.fr |
| Azouvi | Philippe | philippe.azouvi@rpc.aphp.fr |
| Azzolini | Maria Luisa | [azzolini.marialuisa@hsr.it](mailto:azzolini.marialuisa@hsr.it) |
| Bartels | Ronald | Ronald.Bartels@radboudumc.nl |
| Barzó | Pál | pbarzo@gmail.com |
| Beauvais | Romuald | beauvais@arttic.eu |
| Beer | Ronny | ronny.beer@i-med.ac.at |
| Bellander | Bo-Michael | bo-michael.bellander@karolinska.se |
| Belli | Antonio | a.belli@bham.ac.uk |
| Benali | Habib | habib.benali@gmail.com |
| Berardino | Maurizio | maurizio_berardino@fastwebnet.it |
| Beretta | Luigi | beretta.luigi@hsr.it |
| Blaabjerg | Morten | [morten.blaabjerg1@rsyd.dk](mailto:morten.blaabjerg1@rsyd.dk) |
| Bragge | Peter | peter.bragge@monash.edu |
| Brazinova | Alexandra | alexandra.brazinova@gmail.com |
| Brinck | Vibeke | vibeke.brinck@quesgen.com |
| Brooker | Joanne | Joanne.Brooker@monash.edu |
| Brorsson | Camilla | [Camilla.Brorsson@umu.se](mailto:Camilla.Brorsson@umu.se) |
| Buki | Andras | 2saturn@gmail.com |
| Bullinger | Monika | bullinger@uke.de |
| Cabeleira | Manuel | mc916@cam.ac.uk |
| Caccioppola | Alessio | alessio.caccioppola@gmail.com |
| Calappi | Emiliana | [calemy02@yahoo.it](mailto:calemy02@yahoo.it) |
| Calvi | Maria Rosa | [calvi.mariarosa@hsr.it](mailto:calvi.mariarosa@hsr.it) |
| Cameron | Peter | [peter.cameron@med.monash.edu.au](mailto:peter.cameron@med.monash.edu.au) |
| Carbayo Lozano | Guillermo | guillermobilbo@gmail.com |
| Carbonara | Marco | marco.carbonara@gmail.com |
| Castaño-León | Ana M. | [ana.maria.castano.leon@gmail.com](mailto:ana.maria.castano.leon@gmail.com) |
| Cavallo | Simona | cavallosimona1@gmail.com |
| Chevallard | Giorgio | [giorgio.chevallard@ospedaleniguarda.it](mailto:giorgio.chevallard@ospedaleniguarda.it) |
| Chieregato | Arturo | [arturo.chieregato@ospedaleniguarda.it](mailto:arturo.chieregato@ospedaleniguarda.it) |
| Citerio | Giuseppe | giuseppe.citerio@unimib.it |
| Clusmann | Hans | hclusmann@ukaachen.de |
| Coburn | Mark Steven | mark.coburn@ukbonn.de |
| Coles | Jonathan | jpc44@wbic.cam.ac.uk |
| Cooper | Jamie D. | jamie.cooper@monash.edu |
| Correia | Marta | Marta.Correia@mrc-cbu.cam.ac.uk |
| Čović | Amra | amra.covic@med.uni-goettingen.de |
| Curry | Nicola | [nicola.curry@ouh.nhs.uk](mailto:nicola.curry@ouh.nhs.uk) |
| Czeiter | Endre | endre.czeiter@gmail.com |
| Czosnyka | Marek | mc141@medschl.cam.ac.uk |
| Dahyot-Fizelier | Claire | c.dahyot-fizelier@chu-poitiers.fr |
| Dark | Paul | paul.m.dark@manchester.ac.uk |
| Dawes | Helen | hdawes@brookes.ac.uk |
| De Keyser | Véronique | [veronique.dekeyser@uza.be](mailto:veronique.dekeyser@uza.be) |
| Degos | Vincent | [vincent.degos@aphp.fr](mailto:vincent.degos@aphp.fr) |
| Della Corte | Francesco | dellacorte.f@gmail.com |
| den Boogert | Hugo | Hugo.denBoogert@radboudumc.nl |
| Depreitere | Bart | bart.depreitere@uzleuven.be |
| Đilvesi | Đula | [djuladjilvesi@gmail.com](mailto:djuladjilvesi@gmail.com) |
| Dixit | Abhishek | ad825@cam.ac.uk |
| Donoghue | Emma | emma.donoghue@monash.edu |
| Dreier | Jens | jens.dreier@charite.de |
| Dulière | Guy-Loup | glduliere@gmail.com |
| Ercole | Ari | ae105@cam.ac.uk |
| Esser | Patrick | pesser@brookes.ac.uk |
| Ezer | Erzsébet | ezererzsebet@yahoo.com |
| Fabricius | Martin | fabricius@dadlnet.dk |
| Feigin | Valery L. | [valery.feigin@aut.ac.nz](mailto:valery.feigin@aut.ac.nz) |
| Foks | Kelly | k.foks@erasmusmc.nl |
| Frisvold | Shirin | Shirin.Kordasti@unn.no |
| Furmanov | Alex | alexpuil@yahoo.com |
| Gagliardo | Pablo | pablog@fivan.org |
| Galanaud | Damien | galanaud@gmail.com |
| Gantner | Dashiell | dashiell.gantner@monash.edu |
| Gao | Guoyi | gao3@sina.com |
| George | Pradeep | george@incf.org |
| Ghuysen | Alexandre | [A.Ghuysen@chu.ulg.ac.be](mailto:A.Ghuysen@chu.ulg.ac.be) |
| Giga | Lelde | lelde.giga@inbox.lv |
| Glocker | Ben | [b.glocker@imperial.ac.uk](mailto:b.glockert@imperial.ac.uk) |
| Golubović | Jagoš | [jagosgolubovic@gmail.com](mailto:jagosgolubovic@gmail.com) |
| Gomez | Pedro A. | [pagolopez@gmail.com](mailto:pagolopez@gmail.com) |
| Gratz | Johannes | johannes.gratz@meduniwien.ac.at |
| Gravesteijn | Benjamin | b.gravesteijn@erasmusmc.nl |
| Grossi | Francesca | francesca.grossi@libero.it |
| Gruen | Russell L. | [russell.gruen@anu.edu.au](mailto:russell.gruen@anu.edu.au) |
| Gupta | Deepak | drdeepakgupta@gmail.com |
| Haagsma | Juanita A. | j.haagsma@erasmusmc.nl |
| Haitsma | Iain | i.haitsma@erasmusmc.nl |
| Helbok | Raimund | Raimund.Helbok@tirol-kliniken.at |
| Helseth | Eirik | EHELSETH@ous-hf.no |
| Horton | Lindsay | [lindsay.horton@stir.ac.uk](mailto:lindsay.horton@stir.ac.uk) |
| Huijben | Jilske | [j.a.huijben@erasmusmc.nl](mailto:j.a.huijben@erasmusmc.nl) |
| Hutchinson | Peter J. | pjah2@cam.ac.uk |
| Jacobs | Bram | [b.jacobs@umcg.nl](mailto:b.jacobs@umcg.nl) |
| Jankowski | Stefan | Stefan.Jankowski@sth.nhs.uk |
| Jarrett | Mike | mike.jarrett@quesgen.com |
| Jiang | Ji-yao | [jiyaojiang@126.com](mailto:jiyaojiang@126.com) |
| Johnson | Faye | faye.johnson@live.co.uk |
| Jones | Kelly | [kejones@aut.ac.nz](mailto:kejones@aut.ac.nz) |
| Karan | Mladen | mladjokaran@gmail.com |
| Kolias | Angelos G. | angeloskolias@gmail.com |
| Kompanje | Erwin | [erwinkompanje@me.com](mailto:erwinkompanje@me.com) |
| Kondziella | Daniel | Daniel.Kondziella@regionh.dk |
| Kornaropoulos | Evgenios | ek481@cam.ac.uk |
| Koskinen | Lars-Owe | [Lars-Owe.Koskinen@umu.se](mailto:Lars-Owe.Koskinen@umu.se) |
| Kovács | Noémi | kovacs.noemi@pte.hu |
| Lagares | Alfonso | algadoc@yahoo.com |
| Lanyon | Linda | lindal@incf.org |
| Laureys | Steven | [steven.laureys@ulg.ac.be](mailto:steven.laureys@ulg.ac.be) |
| Lecky | Fiona | f.e.lecky@sheffield.ac.uk |
| Ledoux | Didier | dledoux@chu.ulg.ac.be |
| Lefering | Rolf | Rolf.Lefering@uni-wh.de |
| Legrand | Valerie | Valerie.Legrand@iconplc.com |
| Lejeune | Aurelie | aurelie.lejeune@chru-lille.fr |
| Levi | Leon | llevi@rambam.health.gov.il |
| Lightfoot | Roger | Roger.Lightfoot@uhs.nhs.uk |
| Lingsma | Hester | h.lingsma@erasmusmc.nl |
| Maas | Andrew I.R. | [andrew.maas@uza.be](mailto:andrew.maas@uza.be) |
| Maegele | Marc | Marc.Maegele@t-online.de |
| Majdan | Marek | [mmajdan@truni.sk](mailto:mmajdan@truni.sk) |
| Manara | Alex | Alex.Manara@nbt.nhs.uk |
| Manley | Geoffrey | ManleyG@ucsf.edu |
| Maréchal | Hugues | Hugues.Marechal@chrcitadelle.be |
| Martino | Costanza | costmartino74@gmail.com |
| Mattern | Julia | Julia.Mattern@med.uni-heidelberg.de |
| McMahon | Catherine | Catherine.McMahon@thewaltoncentre.nhs.uk |
| Melegh | Béla | bela.melegh@aok.pte.hu |
| Menon | David | dkm13@cam.ac.uk |
| Menovsky | Tomas | [tomas.menovsky@uza.be](mailto:tomas.menovsky@uza.be) |
| Mikolic | Ana | a.mikolic@erasmusmc.nl |
| Misset | Benoit | Benoit.Misset@chuliege.be |
| Muraleedharan | Visakh | visakh@incf.org |
| Murray | Lynnette | lynnette.murray@monash.edu |
| Nair | Nandesh | [nandesh.nair@uza.be](mailto:nandesh.nair@uza.be) |
| Negru | Ancuta | [negruancu@gmail.com](mailto:negruancu@gmail.com) |
| Nelson | David | david.nelson@karolinska.se |
| Newcombe | Virginia | vfjn2@cam.ac.uk |
| Nieboer | Daan | [d.nieboer@erasmusmc.nl](mailto:d.nieboer@erasmusmc.nl) |
| Nyirádi | József | nyiradi.jozsef@pte.hu |
| Oresic | Matej | [matej.oresic@oru.se](mailto:matej.oresic@oru.se) |
| Ortolano | Fabrizio | [lupeda@gmail.com](mailto:lupeda@gmail.com) |
| Otesile | Olubukola | o.otesile@sheffield.ac.uk |
| Palotie | Aarno | aarno.palotie@helsinki.fi |
| Parizel | Paul M. | paul.parizel@uantwerpen.be |
| Payen | Jean-François | Jean-Francois.Payen@ujf-grenoble.fr |
| Perera | Natascha | perera@arttic.eu |
| Perlbarg | Vincent | vincent.perlbarg@gmail.com |
| Persona | Paolo | ppersona75@gmail.com |
| Peul | Wilco | W.C.Peul@lumc.nl |
| Piippo-Karjalainen | Anna | anna.piippo@hus.fi |
| Pirinen | Matti | matti.pirinen@helsinki.fi |
| Pisica | Dana | d.pisica@erasmusmc.nl |
| Ples | Horia | horia.ples@neuromed.ro |
| Polinder | Suzanne | s.polinder@erasmusmc.nl |
| Pomposo | Inigo | inigo.pomposo@osakidetza.net |
| Posti | Jussi P. | [jussi.posti@tyks.fi](mailto:jussi.posti@tyks.fi) |
| Puybasset | Louis | louis.puybasset@aphp.fr |
| Rădoi | Andreea | [aradoi@neurotrauma.net](mailto:aradoi@neurotrauma.net) |
| Ragauskas | Arminas | telematics@ktu.lt |
| Raj | Rahul | [rahul.raj@hus.fi](mailto:rahul.raj@hus.fi) |
| Rambadagalla | Malinka | malinka.rambadagalla@gmail.com |
| Rehorčíková | Veronika | rehorcikova@gmail.com |
| Retel Helmrich | Isabel | i.retelhelmrich@erasmusmc.nl |
| Rhodes | Jonathan | jrhodes1@staffmail.ed.ac.uk |
| Richardson | Sylvia | sylvia.richardson@mrc-bsu.cam.ac.uk |
| Richter | Sophie | sr773@cam.ac.uk |
| Ripatti | Samuli | samuli.ripatti@helsinki.fi |
| Rocka | Saulius | saulius.rocka@mf.vu.lt |
| Roe | Cecilie | e.c.t.roe@medisin.uio.no |
| Roise | Olav | olav.roise@medisin.uio.no |
| Rosand | Jonathan | jrosand@partners.org |
| Rosenfeld | Jeffrey | J.Rosenfeld@alfred.org.au |
| Rosenlund | Christina | chrisstenrose@gmail.com |
| Rosenthal | Guy | [rosenthalg@hadassah.org.il](mailto:rosenthalg@hadassah.org.il) |
| Rossaint | Rolf | RRossaint@ukaachen.de |
| Rossi | Sandra | sandrarossi0@gmail.com |
| Rueckert | Daniel | d.rueckert@imperial.ac.uk |
| Rusnák | Martin | mrusnak@igeh.org |
| Sahuquillo | Juan | sahuquillo@neurotrauma.net |
| Sakowitz | Oliver | oliver.sakowitz@gmail.com |
| Sanchez-Porras | Renan | renan_md@hotmail.com |
| Sandor | Janos | sandor.janos@sph.unideb.hu |
| Schäfer | Nadine | Nadine.Schaefer@uni-wh.de |
| Schmidt | Silke | silke.schmidt@uni-greifswald.de |
| Schoechl | Herbert | Herbert.Schoechl@auva.at |
| Schoonman | Guus | g.schoonman@tsz.nl |
| Schou | Rico Frederik | [rico@mymedic.dk](mailto:rico@mymedic.dk) |
| Schwendenwein | Elisabeth | elisabeth.schwendenwein@meduniwien.ac.at |
| Sewalt | Charlie | c.sewalt@erasmusmc.nl |
| Skandsen | Toril | [toril.skandsen@ntnu.no](mailto:toril.skandsen@ntnu.no) |
| Smielewski | Peter | ps10011@cam.ac.uk |
| Sorinola | Abayomi | sorinola_abayomi@hotmail.com |
| Stamatakis | Emmanuel | [eas46@cam.ac.uk](mailto:eas46@cam.ac.uk) |
| Stanworth | Simon | simon.stanworth@nhsbt.nhs.uk |
| Kowark | Ana | akowark@ukaachen.de |
| Stevens | Robert | rstevens@jhmi.edu |
| Stewart | William | [william.stewart@glasgow.ac.uk](mailto:william.stewart@glasgow.ac.uk) |
| Steyerberg | Ewout W. | [e.w.steyerberg@lumc.nl](mailto:e.w.steyerberg@lumc.nl) |
| Stocchetti | Nino | stocchet@policlinico.mi.it |
| Sundström | Nina | [Nina.Sundstrom@vll.se](mailto:Nina.Sundstrom@vll.se) |
| Takala | Riikka | [riikka.takala@tyks.fi](mailto:riikka.takala@tyks.fi) |
| Tamás | Viktória | tamas.viktoria@pte.hu |
| Tamosuitis | Tomas | tomas.tamosuitis@kaunoklinikos.lt |
| Taylor | Mark Steven | marktrnava@gmail.com |
| Te Ao | Braden | braden.teao@aut.ac.nz |
| Tenovuo | Olli | olli.tenovuo@tyks.fi |
| Theadom | Alice | alice.theadom@aut.ac.nz |
| Thomas | Matt | Matt.Thomas@nbt.nhs.uk |
| Tibboel | Dick | d.tibboel@erasmusmc.nl |
| Timmers | Marjolijn | mtimmers@hotmail.com |
| Tolias | Christos | christos.tolias@nhs.net |
| Trapani | Tony | tony.trapani@monash.edu |
| Tudora | Cristina Maria | cristina.tudora@neuromed.ro |
| Unterberg | Andreas | Andreas.Unterberg@med.uni-heidelberg.de |
| Vajkoczy | Peter | Peter.Vajkoczy@charite.de |
| Valeinis | Egils | Egils.Valeinis@latnet.lv |
| Vallance | Shirley | S.Vallance@alfred.org.au |
| Vámos | Zoltán | azozoka@gmail.com |
| Van der Jagt | Mathieu | m.vanderjagt@erasmusmc.nl |
| van der Naalt | Joukje | j.van.der.naalt@umcg.nl |
| Van der Steen | Gregory | gregory@webstone.be |
| van Dijck | Jeroen T.J.M. | [j.van.dijck@haaglandenmc.nl](mailto:j.van.dijck@haaglandenmc.nl) |
| van Essen | Thomas A. | T.A.van_Essen@lumc.nl |
| Van Hecke | Wim | wim.vanhecke@icometrix.com |
| van Heugten | Caroline | Caroline.vanheugten@maastrichtuniversity.nl |
| Van Praag | Dominique | [dominique.vanpraag@uza.be](mailto:dominique.vanpraag@uza.be) |
| Van Veen | Ernest | e.vanveen.1@erasmusmc.nl |
| van Wijk | Roel | roel-van-wijk@ziggo.nl |
| Vande Vyvere | Thijs | thijs.vandevyvere@icometrix.com |
| Vargiolu | Alessia | neurorianimazione@hsgerardo.org |
| Vega | Emmanuel | emmanuel.vega@chru-lille.fr |
| Velt | Kimberley | [k.velt@erasmusmc.nl](mailto:k.velt@erasmusmc.nl) |
| Verheyden | Jan | jan.verheyden@icometrix.com |
| Vespa | Paul M. | [PVespa@mednet.ucla.edu](mailto:aarno.palotie@helsinki.fi) |
| Vik | Anne | [anne.vik@ntnu.no](mailto:anne.vik@ntnu.no) |
| Vilcinis | Rimantas | rimantas.vilcinis@kaunoklinikos.lt |
| Volovici | Victor | v.volovici@erasmusmc.nl |
| von Steinbüchel | Nicole | nvsteinbuechel@med.uni-goettingen.de |
| Voormolen | Daphne | [d.voormolen@erasmusmc.nl](mailto:d.voormolen@erasmusmc.nl) |
| Vulekovic | Petar | pvulekovic@gmail.com |
| Wang | Kevin K.W. | kawangwang17@gmail.com |
| Wiegers | Eveline | e.wiegers@erasmusmc.nl |
| Williams | Guy | gbw1000@wbic.cam.ac.uk |
| Wilson | Lindsay | l.wilson@stir.ac.uk |
| Winzeck | Stefan | sw742@cam.ac.uk |
| Wolf | Stefan | stefan.wolf@charite.de |
| Yang | Zhihui | [zhihuiyang@ufl.edu](mailto:zhihuiyang@ufl.edu) |
| Ylén | Peter | peter.ylen@vtt.fi |
| Younsi | Alexander | alexander.younsi@med.uni-heidelberg.de |
| Zeiler | Frederick A. | [umzeiler@myumanitoba.ca](mailto:umzeiler@myumanitoba.ca) |
| Ziverte | Agate | agate.ziverte@inbox.lv |
| Zoerle | Tommaso | tommaso.zoerle@policlinico.mi.it |

**Additional Methods**

*Instrumental variable analysis*

The main analysis associates center-level treatment strategies to functional outcome to deduce effectiveness. This natural experiment, in which patients are ‘allocated’ to one or another treatment strategy based on where the accident occurred, leads to considerable reduction of (unmeasured) confounding because patients are brought to hospitals without knowledge of neurosurgical treatment preference. This is an instrumental variable (IV) analysis, a quasi-experimental approach, where the IV center ‘allocates’ patients to be exposed to different likelihoods of receiving primary decompressive craniectomy (DC). IV analysis is less biased by (unmeasured) confounding by indication and is the preferred analytical method in observational studies on acute neurosurgical decisions in traumatic brain injury.^1-3^

The IV approach accounts for unmeasured confounding for which two verifiable assumptions must be met: 1) the instrument is associated with the intervention, and 2) the instrument is not independently associated with the outcome.^4^ The first assumption was addressed using the median odds ratio (MOR), which quantifies the between-center primary DC variation that is not explained by other factors in the model or attributable to chance. A fixed-effects logistic regression model was compared to a model with random intercept for center (with age, GCS score, pupil reactivity, midline shift, concomitant contusion and hematoma size as fixed-effects in both models) with the likelihood ratio test to determine the significance of between-center variation and the partial F statistic as a measure for explained variance.^3^ The second assumption was addressed by comparing baseline prognosis (CRASH-CT score) across the levels of the IV. Moreover, associations between the IV and measured confounders were checked by calculating Spearman’s correlation coefficients between the IV (center preference) and predicted probabilities of unfavorable outcome.

For the IV analyses, the (common) ORs of primary and secondary endpoints are presented as an incremental increase from the 25th percentile to the 75th percentile (the interquartile range [IQR]) of the adjusted primary DC probabilities (treatment preference). The resulting adjusted OR indicates the odds of a more favorable outcome for patient in a hospital at the 25th percentile compared to a patient in a hospital at the 75th percentile on the range of the center surgical preference. To minimize the influence of chance, centers with data on at least 10 patients were included in the primary IV analyses which was a deviation from our protocol.^5^ The protocol stated to include centers contributing at least 15 patients but that resulted in considerably smaller sample. The study protocol was published before patient enrollment.^5^

*Sensitivity analyses*

As sensitivity IV analysis, the instrument was compared with prior collected data on preference for a pre-emptive and/or routine approach with regard to DC when evacuating ASDH from the provider profiling of the CENTER-TBI study.^6^

Additional analyses were performed with primary DC defined at patient-level (exposed to intervention, yes/no), unadjusted, with multivariable regression and propensity score matching (PSM) and restricting to the BTF-guideline subgroup. The propensity of being exposed to the intervention was computed using multivariable logistic regression with primary DC as dependent variable. PSM was used to match exposed patients with non-exposed patients. The maximum difference between propensity scores was set at 0.10 (the caliper) using a nearest neighbor approach in 1:1 balance. For both the propensity score model and the covariable-adjusted model, the aforementioned confounding variables of the primary analysis were considered independent variables. We used random-effects proportional odds models with center as the clustering variable for all patient-level analyses, except for the QOLIBRI analysis in which we used linear regression.

**Additional Results**

*Quantification of practice variation and instrumental variable assumptions*

The instrument was consequently strongly associated with the intervention under study (partial F statistic 28; Supplemental Table 2). Further, correlations between the instruments and measured confounders were small (Spearman’s Rho correlation -0.17; eTable 4), meaning that despite differences in baseline characteristics, the predicted 6-month functional outcome of the CRASH-CT score was similar across centers. The *a priori* reported policy of a low threshold for primary DC was the strongest predictor of observed primary DC, i.e. over and above all other predictors (OR 8.8, 95% CI 1.9 – 40.0, as compared to e.g. variable ‘two nonreactive pupils’ with OR 1.4, 95% CI 0.5 – 4.0), confirming that primary DC rates reflect center treatment preferences (Table 1).

The MOR was 2.2 (p = 0.0007) when excluding the two outlying centres (appendix 33).

*Sensitivity analyses*

On a continuous outcome scale in linear regression, higher adjusted primary DC rates per center were not associated with higher mean GOSE scores. For an increase from the 25th to the 75th percentile (i.e. the IQR) of the adjusted probabilities for primary DC, the mean GOSE decreased non-significantly with 0.1 for primary DC (95% CI: -0.2 – 0.1); Supplemental Figure 2). In a similar analysis excluding centers with < 15 patients the mean GOSE increased non-significantly with 0.1 for primary DC (95% CI: -1.1 – 1.4)).

QOLIBRI scores did not differ in unadjusted analysis (beta -3.9, 95% CI -12.5 – 4.7, n = 130) and also not in adjusted analyses (multivariable regression adjusting for age, GCS, pupils, midline shift: beta -3.4, 95% CI -12.1 – 5.4, n = 130).

**Supplemental Table 1. Baseline and treatment characteristics of patients with acute surgery for traumatic acute subdural hematoma, comparing primary decompressive craniectomy and craniotomy**

| **Patient characteristic** | **Treatment (n = 336)** | | | |
| --- | --- | --- | --- | --- |
|  | **Decompressive craniectomy** | **Craniotomy** | **SMD** | **Missing (%)** |
| n | 91 | 245 |  |  |
| Age (median [IQR]) | 49 [35, 62] | 59 [42, 68] | 0.42 | 0 |
| Male sex (%) | 73 (80) | 169 (69) | 0.24 | 0 |
| ASAPS (%) |  |  | 0.40 | 0 |
| Healthy | 51 (56) | 108 (44) |  |  |
| Mild systemic disease | 18 (20) | 88 (36) |  |  |
| Severe systemic disease | 13 (14) | 36 (15) |  |  |
| Threat to life | 1 (1) | 1 (0) |  |  |
| Unknown | 8 (9) | 11 (5) |  |  |
| Injury cause (%) |  |  | 0.33 | 0 |
| Road traffic incident | 29 (33) | 67 (29) |  |  |
| Incidental fall | 44 (49) | 114 (49) |  |  |
| Other non-intentional injury | 2 (2) | 9 (4) |  |  |
| Assault/violence | 7 (8) | 15 (6) |  |  |
| Suicide attempt | 3 (3) | 3 (1) |  |  |
| Hypoxia (%) ^a^ |  |  | 0.36 | 1 |
| No | 66 (75) | 201 (82) |  |  |
| Definite | 12 (14) | 12 (5) |  |  |
| Suspect | 6 (7) | 10 (4) |  |  |
| Unknown | 4 (5) | 21 (9) |  |  |
| Hypotension (%) ^b^ |  |  | 0.10 | 1 |
| No | 76 (87) | 205 (84) |  |  |
| Definite | 3 (4) | 12 (5) |  |  |
| Suspect | 4 (5) | 10 (4) |  |  |
| Unknown | 5 (6) | 17 (7) |  |  |
| Any major extracranial injury (%) ^c^ | 48 (53) | 89 (36) | 0.34 | 0 |
| GCS (median [IQR]) | 4 [3, 8] | 7 [3, 13] | 0.50 | 5 |
| GCS motor (median [IQR]) | 1 [1, 4] | 4 [1, 6] | 0.53 | 2 |
| Pupils (%) |  |  | 0.26 | 4 |
| Both reacting | 53 (59) | 162 (70) |  |  |
| One reacting | 13 (14) | 31 (13) |  |  |
| Both unreacting | 24 (27) | 38 (17) |  |  |
| Any focal neurological deficit (%) |  |  | 0.39 | 0 |
| No | 33 (36) | 124 (51) |  |  |
| Yes | 12 (13) | 43 (18) |  |  |
| Unknown | 46 (51) | 77 (32) |  |  |
| Anti-coagulants or platelet aggregation inhibitors (%) |  |  | 0.31 | 0 |
| No | 71 (78) | 164 (67) |  |  |
| Anti-coagulants | 5 (6) | 28 (12) |  |  |
| Platelet inhibitors | 8 (9) | 30 (12) |  |  |
| Both | 0 (0) | 3 (1) |  |  |
| Unknown | 7 (8) | 19 (8) |  |  |
| Any neuroworsening before surgery (%) | 25 (28) | 71 (29) | 0.05 | 0 |
| Total volume of ASDH (cm3, median [IQR]) | 64 [34, 97] | 49 [20, 83] | 0.32 | 32 |
| CT large ASDH (%) ^d^ | 69 (76) | 183 (75) | 0.03 | 0 |
| CT midline shift (%) ^e^ | 79 (87) | 208 (85) | 0.06 | 0 |
| CT contusion (%) |  |  | 0.28 | 0 |
| No | 28 (31) | 106 (43) |  |  |
| Small | 38 (42) | 94 (38) |  |  |
| Large | 21 (23) | 39 (16) |  |  |
| Unknown | 3 (3) | 6 (2) |  |  |
| CT SAH (%) |  |  | 0.38 | 0 |
| No | 23 (25) | 94 (38) |  |  |
| Basal | 6 (7) | 20 (8) |  |  |
| Cortical | 38 (42) | 97 (40) |  |  |
| Basal and Cortical | 24 (26) | 34 (14) |  |  |
| CT basal cisterns absent/compressed (%) | 50 (56) | 106 (44) | 0.24 | 1 |
| Mean predicted 6-month unfavorable outcome (GOS score ≤ 3, %, median [IQR]) ^f^ | 71 [59, 86] | 74 [50, 87] | 0.11 | 0 |
| Acute surgery characteristics |  |  |  |  |
| Surgery type |  | Na |  | 9 |
| Hemi-craniectomy | 79 (87) |  |  |  |
| Bifrontal-craniectomy | 4 (5) |  |  |  |
| Time from injury to surgery (minutes, median [IQR]) | 210 [131, 303] | 246 [165, 420] | 0.11 | 1 |
| ICP |  |  |  | 42 |
| Day 1 (median [IQR]) | 9 [5, 19] | 7 [3, 15] | 0.46 |  |
| Max day 1 (median [IQR]) | 12 [2, 27] | 7 [0, 16] | 0.70 |  |

Abbreviation: AIS, Abbreviated Injury Scale; ASAPS, American Society of Anesthesiologists classification system; ASDH, acute subdural hematoma; CPP, cerebral perfusion pressure; GCS, Glasgow Coma Scale; GOS, Glasgow Outcome Scale (5-point); ICP, intracranial pressure; IQR, interquartile range; SAH, subarachnoid hemorrhage; SMD, standardized mean difference.

^a^ Second insult during the pre-hospital or ER phase, defined as PaO2 < 8 kPa (60 mmHg)/SaO2 < 90%. “Suspected” was scored if the patient did not have documented hypoxia by PaO2 or SaO2, but there was a clinical suspicion, as evidenced by for example cyanosis, apnea or respiratory distress.

^b^ Second insult during the pre-hospital or ER phase, defined as systolic BP < 90 mmHg. "Suspected" was scored if the patient did not have a documented blood pressure, but was reported to be in shock or have an absent brachial pulse (not related to injury of the extremity).

^c^ AIS ≥ 3

^d^ Large is defined qualitatively by the treating neurosurgeon and corresponded to a size larger than 25 cm³.

^e^ Midline shift present is classified as being more than 5 mm.

^f^ TBI severity as summarized in predicted unfavorable outcome, proportion with a Glasgow Outcome Scale score ≤ 3, based on CRASH-CT variables.

**Supplemental Table 2. Characteristics of primary decompressive craniectomy**

**A. Reasons for DC**

| **Patient characteristic** |  |  |
| --- | --- | --- |
|  |  | Missing |
| n | 91 |  |
| Reason for DC (%) |  | 10 |
| Pre-emptive approach to treatment of (suspected) raised ICP (not last resort) | 25 (31) |  |
| Raised ICP, refractory to medical management (last resort) | 17 (21) |  |
| ICP not monitored, but CT evidence of raised ICP | 21 (26) |  |
| Not directly planned, but decided on because of intra-operative brain swelling | 14 (17) |  |
| Routinely performed with every ASDH or contusion evacuation | 4 (5) |  |
| Development of cerebral infarction | 1 (1) |  |

Abbreviation: ASDH, acute subdural hematoma; DC, decompressive craniectomy; ICP, intracranial pressure.

**B. ICP values over time**

|  | Pre-emptive approach to treatment of (suspected) raised ICP (not last resort) | Raised ICP, refractory to medical management (last resort) | ICP not monitored, but CT evidence of raised ICP | Not directly planned, but decided on because of intra-operative brain swelling | Routinely performed with every ASDH or Contusion evacuation | Development of cerebral infarction | p |
| --- | --- | --- | --- | --- | --- | --- | --- |
| n | 14 | 8 | 10 | 4 | 2 | 1 |  |
| ICP day 1 (median [IQR]) | 10 [4, 17] | 8 [7, 28] | 21 [10, 32] | 5 [4, 7] | 5 [4, 6] | 9 [9, 9] | 0.34 |
| ICP day 2 (median [IQR]) | 12 [5, 17] | 16 [11, 21] | 18 [13, 25] | 12 [10, 12] | 7 [6, 8] | 9 [9, 9] | 0.29 |
| ICP max day 1 (median [IQR]) | 10 [0, 17] | 16 [10, 32] | 26 [14, 48] | 10 [0, 12] | 5 [4, 6] | 11 [11, 11] | 0.15 |
| ICP max day 2 (median [IQR]) | 18 [3, 26] | 18 [14, 25] | 22 [18, 30] | 15 [0, 15] | 10 [8, 11] | 11 [11, 11] | 0.46 |

Abbreviation: ICP, intracranial pressure; IQR, interquartile range.

**Supplemental Table 3. Hospital course, complications and follow-up of patients with acute surgery for traumatic acute subdural hematoma, comparing primary decompressive craniectomy and craniotomy**

|  | **Treatment (n = 336)** | | | |
| --- | --- | --- | --- | --- |
|  | **Decompressive craniectomy** | **Craniotomy** | **SMD** | **Missing (%)** |
| n | 91 | 245 |  |  |
| Length of hospital stay (median [IQR]) | 38 [24, 67] | 18 [10, 36] | 0.42 | 0 |
| Any neuroworsening after surgery (%) ^a^ | 48 (53) | 100 (41) | 0.23 | 0 |
| Progression on CT (%) ^b^ | 36 (40) | 83 (34) | 0.10 | 1 |
| Increase in initial lesion | 26 (72) | 50 (60) |  |  |
| Development of new lesion | 10 (28) | 32 (39) |  |  |
| Secondary surgery (%) | 25 (27) | 43 (18) |  | 27 |
| DC | 14 (56) | 12 (28) |  |  |
| ASDH | 9 (36) | 23 (53) |  |  |
| Contusion/ICH | 2 (8) | 8 (190 |  |  |
| Time injury to secondary or delayed surgery (min, median [IQR]) | 795 [466, 29090] | 1210 [330, 4310] |  |  |
| ICP monitor (%) | 79 (87) | 157 (64) | 0.55 | 0.0 |
| ICP (median [IQR]) | 12 [9, 17] | 12 [8, 15] | 0.23 | 32 |
| CPP (median [IQR]) | 74 [70, 77] | 73 [67, 77] | 0.02 | 32 |
| TIL (median [IQR]) | 8 [6, 12] | 3 [1, 7] | 1.01 | 2 |
| Extracranial surgery (%) | 25 (28) | 44 (18) | 0.23 | 0 |
| Other cranial surgery (during admission, after DC and craniotomy respectively, %) |  |  |  |  |
| Epidural hematoma | 4 (4) | 7 (3) |  |  |
| Ventriculostomy for CSF drainage | 12 (13) | 23 (9) |  |  |
| Chronic subdural hematoma | 1 (1) | 0 (0) |  |  |
| Cranioplasty | 23 (25) | 12 (5) |  |  |
| CSF shunt | 10 (11) | 12 (5) |  |  |
| Complications (requiring treatment) |  |  |  | 0 |
| Delayed intracranial hematoma/seroma (%) | 21 (23) | 40 (16) | 0.17 |  |
| Raised ICP (%) | 50 (55) | 84 (34) | 0.43 |  |
| Meningitis (%) | 11 (12) | 16 (7) | 0.19 |  |
| Seizure (%) | 11 (12) | 31 (13) | 0.02 |  |
| Other intracranial complication %) | 9 (10) | 14 (6) | 0.16 |  |
| Ventilator-associated pneumonia (%) | 24 (26) | 32 (13) | 0.34 |  |
| Cardiac arrest (%) | 7 (8) | 17 (7) | 0.03 |  |
| Respiratory (%) | 21 (23) | 50 (20) | 0.07 |  |
| Cardiovascular (%) | 2 (2) | 11 (5) | 0.13 |  |
| Metabolic (%) | 10 (11) | 12 (5) | 0.23 |  |
| CRBSI (%) | 4 (4) | 7 (3) | 0.08 |  |
| Deep venous thrombosis (%) | 4 (4) | 7 (3) | 0.08 |  |
| Pulmonary embolus (%) | 3 (3) | 5 (2) | 0.08 |  |
| Pressure sores (decubitus) (%) | 10 (11) | 15 (6) | 0.18 |  |
| Urinary tract infection (%) | 8 (8) | 22 (9) | 0.01 |  |
| Other systemic complication (%) | 11 (12) | 31 (13) | 0.02 |  |
| Discharge destination |  |  | 0.36 | 38 |
| Other hospital | 12 (25) | 49 (32) |  |  |
| Rehabilitation unit | 22 (46) | 61 (40) |  |  |
| Nursing home | 5 (10) | 10 (7) |  |  |
| Home | 5 (10) | 27 (18) |  |  |
| Other | 4 (8) | 5 (3) |  |  |
| Treatment during follow-up (after discharge) |  |  |  | 0 |
| Hydrocephalus | 7 (8) | 2 (1) |  |  |
| Chronic subdural hematoma | 2 (2) | 4 (2) |  |  |
| Cranioplasty | 14 (15) | 11 (4) |  |  |

Abbreviation: DC, decompressive craniectomy; CRBSI, catheter-related bloodstream infection; CSF, cerebrospinal fluid; ICP, intracranial pressure; IQR, interquartile range; TIL, Therapy Intensity Level.

^a^ Neuroworsening is defined as: a spontaneous decrease in the Glasgow Coma Scale motor score ≥ 2 points (compared with the previous examination), a new loss of pupillary reactivity, development of pupillary asymmetry ≥ 2 mm, and/or deterioration in neurological or CT status sufficient to warrant immediate medical or surgical intervention.

^b^ Progression on the CT scan during the hospital course is defined as an increase in initial lesion and/or the development of a new lesion.

**Supplemental Table 4. Assumptions for instrumental variable analyses**

| *IV assumptions* | *Primary DC* |
| --- | --- |
| Assumption 1: instrument association with intervention |  |
| Partial F statistic | 28.3 |
| Assumption 2: instrument association with prognosis |  |
| Spearman’s Rho correlation with P_Unfavorable_ ^a^ | -0.17 |

Abbreviation: DC, decompressive craniectomy; IV, instrumental variable

^a^ Prognosis as summarized in CT-CRASH score, predicted unfavorable outcome (proportion with a Glasgow Outcome Scale score ≤ 3)

**Supplemental Table 5. Hospital course and outcome across centers with different preferences for primary decompressive craniectomy**

|  | **Treatment preference (observed acute surgery rates)** | | | |  |
| --- | --- | --- | --- | --- | --- |
|  | **Quartile 1 (6 - 12%)** | **Quartile 2 (12 – 19%)** | **Quartile 3 (19 – 26%)** | **Quartile 4 (26 - 67%)** | **SMD** |
| n | 53 | 48 | 51 | 48 |  |
| Any neuroworsening after surgery (%) ^a^ | 23 (43) | 13 (27) | 27 (53) | 32 (67) | 0.45 |
| Progression on CT (%) ^b^ | 22 (42) | 15 (31) | 21 (41) | 21 (44) | 0.13 |
| ICP monitor placement (%) | 44 (83) | 25 (52) | 34 (67) | 37 (77) | 0.38 |
| ICP (median [IQR]) | 12 [8, 15] | 11 [8, 16] | 10 [7, 17] | 12 [7, 16] | 0.10 |
| CPP (median [IQR]) | 68 [61, 73] | 71 [65, 76] | 72 [67, 76] | 74 [69, 76] | 0.07 |
| TIL (median [IQR]) | 5 [2, 8] | 3 [1, 5] | 5 [2, 9] | 6 [3, 11] | 0.53 |
| Length of hospital stay (days, [IQR])) | 17 [10, 35] | 13 [7, 30] | 17 [8, 28] | 24 [6, 46] | 0.24 |
| Length of ICU stay (median [IQR]) | 8 [3, 16] | 11 [3, 18] | 8 [2, 18] | 7 [2, 14] | 0.23 |
| In-hospital mortality (%) | 12 (23) | 11 (23) | 21 (41) | 18 (38) | 0.26 |
| GOSE 6-months (%) |  |  |  |  | 0.53 |
| 1=Dead | 18 (34) | 14 (29) | 21 (41) | 20 (42) |  |
| 2=Vegetative state/3=Lower severe disability | 15 (28) | 13 (27) | 11 (22) | 6 (12) |  |
| 4=Upper severe disability | 3 (6) | 1 (2) | 3 (6) | 5 (10) |  |
| 5=Lower moderate disability | 3 (6) | 10 (21) | 7 (14) | 6 (12) |  |
| 6=Upper moderate disability | 1 (2) | 0 (0) | 2 (4) | 3 (6) |  |
| 7=Lower good recovery | 6 (11) | 7 (15) | 4 (8) | 6 (12) |  |
| 8=Upper good recovery | 7 (13) | 3 (6) | 3 (6) | 2 (4) |  |

Abbreviation: CPP, cerebral perfusion pressure; DC, decompressive craniectomy; GOSE, Glasgow Outcome Scale Extended; ICU, intensive care unit; IQR, interquartile range; SMD, standardized mean difference; TIL, therapy intensity level.

^a^ Neuroworsening is defined as: a spontaneous decrease in the Glasgow Coma Scale motor score ≥ 2 points (compared with the previous examination), a new loss of pupillary reactivity, development of pupillary asymmetry ≥ 2 mm, and/or deterioration in neurological or CT status sufficient to warrant immediate medical or surgical intervention.

^b^ Progression on the CT scan during the hospital course is defined as an increase in initial lesion and/or the development of a new lesion.

**Supplemental Figure 1. Propensity scores distribution of nonmatched cohorts and propensity matched cohorts of primary decompressive craniectomy**

The propensity of being exposed to the intervention was computed using multivariable logistic regression with primary decompressive craniectomy as the dependent variable. Propensity score matching was used to match exposed patients with non-exposed patients. The maximum difference between propensity scores was set at 0·10 (the caliper) using a nearest neighbor approach in 1:1 balance.


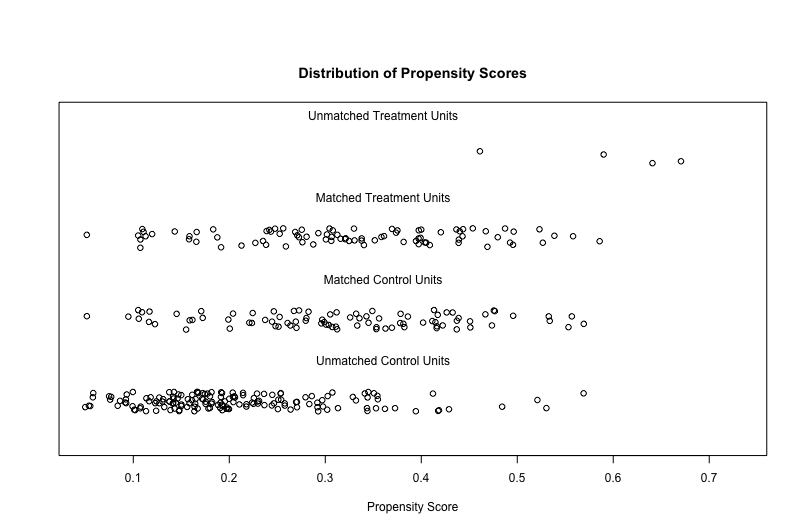


**Supplemental Table 6. Results of sensitivity analyses: comparing analytical methods to adjust for confounding by indication in proportional odds logistic regression models with**

**the Glasgow Outcome Scale Extended score as outcome**

| **Approach** | **Primary DC (common OR 95 % CI, number of patients)** |
| --- | --- |
| Unadjusted model | 0.4 (0.3 – 0.6, n = 336) |
| Covariable adjustment ^a^ | 0.4 (0.2 – 0.6, n = 336) |
| Propensity score matching ^b^ | 0.4 (0.3 – 0.8, n = 174) |
| Instrumental variable ^c^ |  |
| With the cohort with centers < 15 patients excluded | 0.9 (0.5 – 1.5, n = 97) |
| Predefined instrument (in provider profiling) ^d^ | 0.9 (0.4 – 2.2, n = 204) |
| Patients without poor baseline prognosis ^e^ | 0.9 (0.8 – 1.1, n = 106) |

Abbreviation: CI, confidence interval; DC, decompressive craniectomy; OR, odds ratio.

^a^ Model was adjusted for the following confounders: age, GCS, pupillary reactivity, midline shift, concomitant contusion and hematoma size, including a random intercept for center.

^b^ A propensity score was calculated based on the following variables: age, GCS, pupillary reactivity, midline shift, concomitant contusion and hematoma size, including a random intercept for center.

^c^ The adjusted common OR indicates the odds of a more favorable outcome for an increase of the 25th percentile to the 75th percentile of the range in exposure to the regional intervention preferences. In this random effects model, the random intercept for each center represents the unexplained center effect (beyond all factors included in the model, including the instrument treatment preference).

^d^ Centers provided in a questionnaire - before patient enrolment - whether they employ a pre-emptive and/or routine approach with regard to DC when evacuating ASDH. For the current analysis, the threshold for primary DC surgery for ASDH was dichotomized accordingly: ‘Yes’, primary DC routinely/pre-emptively versus ‘No’, no primary DC routinely/pre-emptively were compared.

The association could not be estimated on the cohort with centers providing 15 patients, because there were no ‘routing/pre-emptive centers’. Instead, the cohort with centers providing at least 10 patients were used.

^e^ This analysis is performed without patients that had a CRASH-CT score within the fourth quartile (ie, higher than 85% predicted unfavorable outcome [proportion with a Glasgow Outcome Scale score ≤ 3]).

**Supplemental Table 7. Unadjusted patient-level analysis**

|  | **Patient-level analyses** | | | |
| --- | --- | --- | --- | --- |
| **Outcome** | **Primary DC (n = 91)** | **Craniotomy (n = 245)** | **Effect variable** | **Unadjusted value (95% CI)** |
|  |  |  |  |  |
| Primary outcome: GOSE at 6 months (median [IQR]) | 3 [1-4] | 4 [1-6] | Common odds ratio | 0.4 (0.3 – 0.6) |
| Secondary outcomes |  |  |  |  |
| In-hospital mortality | 35 (38) | 58 (24) | Odds ratio | 2.0 (1.2 – 3.4) |
| GOSE of 7 or 8 (%) | 4 (4) | 50 (20) | Odds ratio | 0.2 (0.1 – 0.5) |
| GOSE of 5-8 (%) | 18 (20) | 108 (44) | Odds ratio | 0.3 (0.2 – 0.6) |
| GOSE of 4-8 (%) | 26 (29) | 126 (51) | Odds ratio | 0.4 (0.2 – 0.6) |
| QOLIBRI (median [IQR]) at 6 months ^a^ | 69 (60-80) | 76 (60-85) | Beta | -3.9 (-12.5 – 4.7) |

Abbreviation: CI, confidence interval; DC, decompressive craniectomy; GOSE, Glasgow Outcome Scale Extended; IQR, interquartile range; Na, not available; QOLIBRI, Quality of Life after Brain Injury Scale.

^a^ QOLIBRI is a standardized health specific quality of life measure specifically designed for and validated in outcome assesment in patients with brain injury. It is a numerical scale with scores ranging from 0 to 100, with higher scores indicating a better quality of life. The score was available for 19 patients of the primary DC group and 111 of the craniotomy group.

**Supplemental Table 8. Baseline characteristics of propensity matched cohort, comparing primary decompressive craniectomy with craniotomy**

| **Patient characteristic** | **Treatment status (n = 174)** | |  |
| --- | --- | --- | --- |
|  | **Decompressive craniectomy** | **Craniotomy** | **SMD** |
| n | 87 | 87 |  |
| Age (median [IQR]) | 51 [36, 63] | 47 [34, 59] | 0.2 |
| ASAPS (%) |  |  | 0.4 |
| Healthy | 48 (55) | 51 (59) |  |
| Mild systemic disease | 17 (20) | 26 (30) |  |
| Severe systemic disease | 13 (15) | 5 (6) |  |
| Threat to life | 1 (1) | 1 (1) |  |
| Unknown | 8 (9) | 4 (5) |  |
| Hypoxia (%) |  |  | 0.5 |
| No | 65 (75) | 68 (78) |  |
| Definite | 13 (15) | 3 (3) |  |
| Suspect | 5 (6) | 7 (8) |  |
| Unknown | 4 (5) | 9 (10) |  |
| Hypotension (%) |  |  | 0.3 |
| No | 76 (87) | 68 (78) |  |
| Definite | 3 (3) | 5 (6) |  |
| Suspect | 3 (3) | 5 (6) |  |
| Unknown | 5 (6) | 9 (10) |  |
| Any major extracranial injury (%) | 46 (53) | 37 (43) | 0.4 |
| GCS (median [IQR]) | 5 [3, 8] | 4 [3, 7] | 0.2 |
| GCS motor (median [IQR]) | 1 [1, 4] | 1 [1, 4] | 0 |
| Pupils (%) |  |  | 0.2 |
| Both reacting | 54 (62) | 52 (60) |  |
| One reacting | 12 (14) | 12 (14) |  |
| Both unreacting | 21 (24) | 23 (26) |  |
| CT large ASDH (%) | 66 (76) | 60 (69) | 0 |
| CT midline shift measure (mm) | 8 [5, 13] | 8 [4, 13] | 0 |
| CT contusion (%) |  |  | 0.2 |
| No | 28 (32) | 31 (36) |  |
| Small | 37 (43) | 31 (36) |  |
| Large | 19 (22) | 20 (23) |  |
| Unknown | 3 (3) | 5 (6) |  |
| GOSE (%) |  |  | 0.5 |
| 1=Dead | 36 (41) | 23 (26) |  |
| 2=Vegetative state/3=Lower severe disability | 26 (30) | 23 (26) |  |
| 4=Upper severe disability | 8 (9) | 8 (9) |  |
| 5=Lower moderate disability | 12 (14) | 15 (17) |  |
| 6=Upper moderate disability | 1 (1) | 6 (7) |  |
| 7=Lower good recovery | 3 (3) | 7 (8) |  |
| 8=Upper good recovery | 1 (1) | 5 (6) |  |

Abbreviation: ASAPS, American Society of Anesthesiologists classification system; ASDH, acute subdural hematoma; GCS, Glasgow Coma Scale; GOSE, Glasgow Outcome Scale Extended; IQR, interquartile range; SMD, standardized mean difference.

**Supplemental Table 9. Selected baseline characteristics and prognosis across centers with different preferences for primary decompressive craniectomy, excluding centers < 15 patients**

| **Patient characteristic** | **Treatment preference (observed primary DC rates per centre) ^a^** | | |  |
| --- | --- | --- | --- | --- |
|  | Tertile 1 (0 – 22) | Tertile 2 (22 – 45) | Tertile 3 (45 – 67) | SMD |
| n | 42 | 40 | 15 |  |
| Age (median [IQR]) | 62 [56, 68] | 63 [41, 68] | 44 [31, 58] | 0.6 |
| ASAPS (%) |  |  |  | 0.8 |
| Healthy | 25 (60) | 20 (50) | 3 (20) |  |
| Mild systemic disease | 14 (33) | 12 (30) | 8 (53) |  |
| Severe systemic disease | 3 (7) | 7 (18) | 2 (13) |  |
| Threat to life | 0 (0) | 1 (3) | 0 (0) |  |
| Unknown | 0 (0) | 0 (0) | 2 (13) |  |
| Hypoxia (%) ^b^ |  |  |  | 0.8 |
| No | 30 (71) | 35 (88) | 11 (73) |  |
| Definite | 1 (2) | 5 (13) | 2 (13) |  |
| Suspect | 2 (5) | 0 (0) | 2 (13) |  |
| Unknown | 9 (21) | 0 (0) | 0 (0) |  |
| Hypotension (%) ^c^ |  |  |  | 0.6 |
| No | 32 (76) | 38 (95) | 13 (87) |  |
| Definite | 1 (2) | 1 (3) | 0 (0) |  |
| Suspect | 0 (0) | 1 (3) | 1 (7) |  |
| Unknown | 9 (21) | 0 (0) | 1 (7) |  |
| Any major extracranial injury (%) ^d^ | 20 (48) | 20 (50) | 8 (53) | 0.1 |
| GCS baseline (median [IQR]) | 8 [3, 11] | 7 [3, 9] | 5 [4, 13] | 0.2 |
| GCS motor baseline (median [IQR]) | 4 [1, 5] | 1 [1, 5] | 3 [2, 6] | 0.4 |
| Pupils (%) |  |  |  | 0.5 |
| Both reacting | 28 (67) | 23 (58) | 9 (60) |  |
| One reacting | 3 (7) | 7 (18) | 0 (0) |  |
| Both unreacting | 11 (26) | 10 (25) | 6 (40) |  |
| Total volume of ASDH (cm3, median [IQR]) | 61 [33, 97] | 73 [33, 100] | 47 [17, 79] |  |
| CT large ASDH (%) ^f^ | 29 (69) | 34 (85) | 10 (67) | 0.3 |
| CT midline shift (%) ^e^ | 32 (76) | 38 (95) | 14 (93) | 0.4 |
| CT contusion (%) |  |  |  | 0.5 |
| No | 14 (33) | 16 (40) | 3 (20) |  |
| Small | 20 (48) | 20 (50) | 10 (67) |  |
| Large | 8 (19) | 3 (8) | 2 (13) |  |
| Unknown | 0 (0) | 1 (3) | 0 (0) |  |
| CT subarachnoid hemorrhage (%) |  |  |  | 0.8 |
| No | 13 (31) | 9 (23) | 6 (40) |  |
| Basal | 5 (12) | 2 (5) | 0 (0) |  |
| Cortical | 17 (41) | 25 (63) | 3 (20) |  |
| Basal and cortical | 7 (17) | 4 (10) | 6 (40) | 0.2 |
| CT basal cisterns absent/compressed (%) | 18 (43) | 19 (48) | 9 (60) | 0.5 |
| Mean predicted 6-month unfavorable outcome (GOS score ≤ 3, %, median [IQR]) ^g^ | 76 [57, 88] | 83 [64, 91] | 65 [45, 73] | 0.2 |

Abbreviation: AIS, Abbreviated Injury Scale; ASAPS, American Society of Anesthesiologists classification system; ASDH, acute subdural hematoma; DC, decompressive craniectomy; GCS, Glasgow Coma Scale; GOS, Glasgow Outcome Scale (5-point); IQR, interquartile range; IV, instrumental variable; SAH, subarachnoid hemorrhage

^a^ Treatment preference as defined by the case-mix adjusted probability of undergoing acute surgery (as opposed to initial conservative treatment) based on the observed acute surgery rates per centre. This corresponds to the IV status and presented in quartiles of the range of adjusted regional primary DC rates. The first category is less aggressive than the second and the second is less aggressive than the third and so forth. Importantly, the IV analysis used the acute surgery rates as continuous preference, the quartiles are presented for purposes of interpretability of baseline comparability.

^b^ Second insult during the pre-hospital or ER phase, defined as PaO2 < 8 kPa (60 mmHg)/SaO2 < 90%. ‘Suspected’ was scored if the patient did not have documented hypoxia by PaO2 or SaO2, but there was a clinical suspicion, as evidenced by for example cyanosis, apnoea or respiratory distress.

^c^ Second insult during the pre-hospital or ER phase, defined as systolic BP < 90 mmHg. ‘Suspected’ was scored if the patient did not have a documented blood pressure, but was reported to be in shock or have an absent brachial pulse (not related to injury of the extremity)

^d^ AIS ≥ 3

^e^ Large is defined qualitatively by the treating neurosurgeon and corresponded to a size larger than 25 cm³.

^f^ Midline shift present is classified as being more than 5 mm.

^g^ TBI severity as summarized in predicted unfavorable outcome, proportion with a Glasgow Outcome Scale score ≤ 3, based on CRASH-CT variables

**Supplemental Table 10. Hospital course and outcome across centers with different preferences for primary decompressive craniectomy, excluding centers < 15 patients**

|  | **Treatment preference (observed primary DC rates per centre)** | | |  |
| --- | --- | --- | --- | --- |
|  | **Tertile 1 (0 – 22)** | **Tertile 2 (22 – 45)** | **Tertile 3 (45 – 67)** | **SMD** |
| n | 42 | 40 | 15 |  |
| Length of hospital stay (median [IQR]) | 21 [10, 35] | 17 [8, 25] | 41 [21, 66] | 0.3 |
| Length of ICU stay (median [IQR]) | 12 [6, 18] | 9 [2, 17] | 10 [5, 18] | 0.1 |
| ICP monitor (%) | 36 (86) | 25 (62) | 12 (80) | 0.4 |
| ICP (median [IQR]) | 12 [9, 17] | 12 [7, 19] | 12 [10, 16] | 0.1 |
| Any neuroworsening after surgery (%) ^a^ | 17 (40) | 21 (52) | 10 (67) | 0.4 |
| Progression on CT (%) ^b^ | 19 (45) | 18 (45) | 7 (47) | <0.1 |
| In-hospital mortality (%) | 11(26) | 16 (40) | 4 (27) | 0.2 |
| GOSE (%) |  |  |  | 0.8 |
| 1=Dead | 16 (38) | 17 (42) | 5 (33) |  |
| 2=Vegetative state/3=Lower severe disability | 11 (26) | 10 (25) | 3 (20) |  |
| 4=Upper severe disability | 3 (7) | 1 (3) | 3 (20) |  |
| 5=Lower moderate disability | 2 (5) | 5 (13) | 3 (20) |  |
| 6=Upper moderate disability | 1 (2) | 1 (3) | 1 (7) |  |
| 7=Lower good recovery | 5 (12) | 4 (10) | 0 (0) |  |
| 8=Upper good recovery | 4 (10) | 2 (5) | 0 (0) |  |

Abbreviation: DC, decompressive craniectomy; GOSE, Glasgow Outcome Scale Extended; ICU, intensive care unit; IQR, interquartile range

^a^ Neuroworsening is defined as: a spontaneous decrease in the Glasgow Coma Scale motor score ≥ 2 points (compared with the previous examination), a new loss of pupillary reactivity, development of pupillary asymmetry ≥ 2 mm, and/or deterioration in neurological or CT status sufficient to warrant immediate medical or surgical intervention.

^b^  Progression on the CT scan during the hospital course is defined as an increase in initial lesion and/or the development of a new lesion.

**Supplemental Table 11. Primary and secondary outcomes and treatment associations for primary decompressive craniectomy, excluding centers < 15 patients**

| *Outcome* | *Intervention (n = 91)* | *Control (n = 245)* | *Effect parameter* | *Unadjusted value of patient-level analysis (95% CI, n = 336)* | *Adjusted value of center-level analysis (95% CI, n = 97) ^a^* |
| --- | --- | --- | --- | --- | --- |
| Primary outcome: GOSE at 6 months (median [IQR]) | 3 [1-4] | 4 [1-6] | Common odds ratio | 0.4 (0.3 – 0.6) | 0.9 (0.5 – 1.5) |
| Secondary outcomes |  |  |  |  |  |
| In-hospital mortality | 35 (38) | 58 (24) | Odds ratio | 2.0 (1.2 – 3.4) | 1.0 (0.5 – 2.4) |
| GOSE of 7 or 8 (%) | 4 (4) | 50 (20) | Odds ratio | 0.2 (0.1 – 0.5) | 0.5 (0.2 – 1.2) |
| GOSE of 5-8 (%) | 18 (20) | 108 (44) | Odds ratio | 0.3 (0.2 – 0.6) | 1.0 (0.5 – 1.8) |
| GOSE of 4-8 (%) | 26 (29) | 126 (51) | Odds ratio | 0.4 (0.2 – 0.6) | 1.2 (0.5 – 1.7) |
| QOLIBRI (median [IQR]) at 6 months ^b^ | 69 (60-80) | 76 (60-85) | Beta | -3.9 (-12.5 – 4.7) | Na ^c^ |

Abbreviation: CI, confidence interval; GOSE, Glasgow Outcome Scale Extended; IQR, interquartile range; IV, instrumental variable; Na, not available; QOLIBRI, Quality of Life after Brain Injury Scale.

^a^ IV analysis with estimates with from random effect multivariable ordinal regression with the instrument, adjusted probability of undergoing primary DC as treatment variable. Confounding was furthermore addressed by adjusting for the a-priori defined variables age, GCS, pupil reactivity, hematoma size, contusion presence and midline shift. The adjusted common OR indicates the odds of a more favorable outcome for an increase of the 25th percentile to the 75th percentile (IQR) of the range in exposure to the center intervention preferences.

^b^ QOLIBRI is a standardized health specific quality of life measure specifically designed for and validated in outcome assesment in patients with brain injury. It is a numerical scale with scores ranging from 0 to 100, with higher scores indicating a better quality of life. The score was available for 19 patients of the primary DC group and 111 of the craniotomy group.

^c^ The association estimate could not be estimated due to low numbers (no centers with more than 15 patients in the subcohort).

**Supplemental Figure 2. Functional outcome of centers with different probabilities of primary decompressive craniectomy**

Graphical illustration to estimate the incremental effect of more primary decompressive craniectomy. Each circle represents a center with the area being proportional to the number of patients per center. The fitted line is the result of an adjusted random effects linear model with dotted lines reporting the 95% confidence intervals. The mean GOSE decreased non-significantly with 0.1 for primary DC (95% CI: -0.2 – 0.1, n = 200).

**
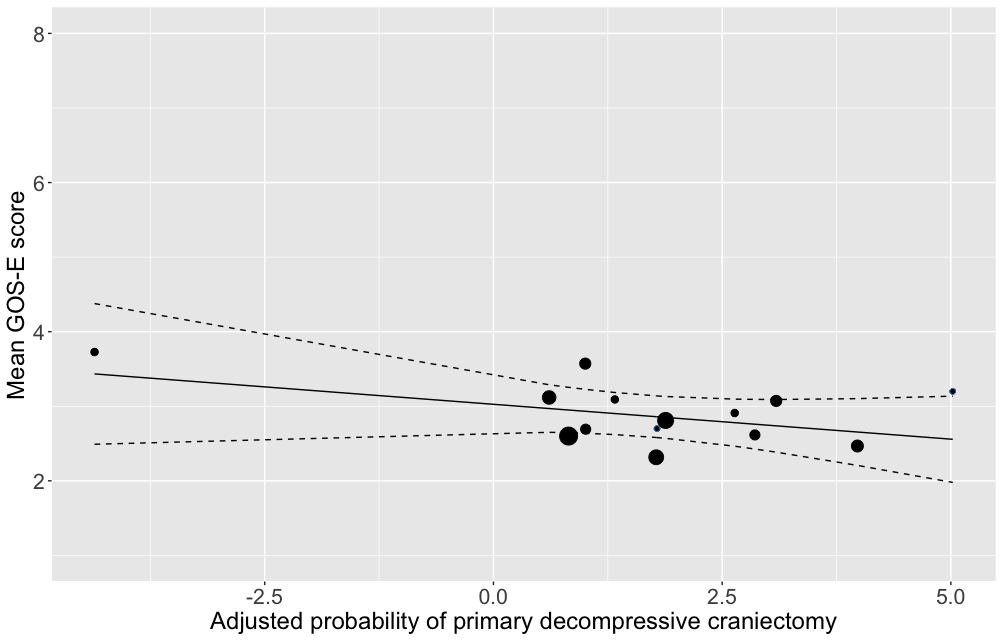
**

**Supplemental Figure 3. Between-centre differences in primary decompressive craniectomy excluding outlying centres.**

The figure shows the case-mix adjusted log odds ratio for primary decompressive craniectomy per centre, excluding two centres considered outliers (see Figure 2B). The MOR is 2.2 (p value = 0·0007).

**
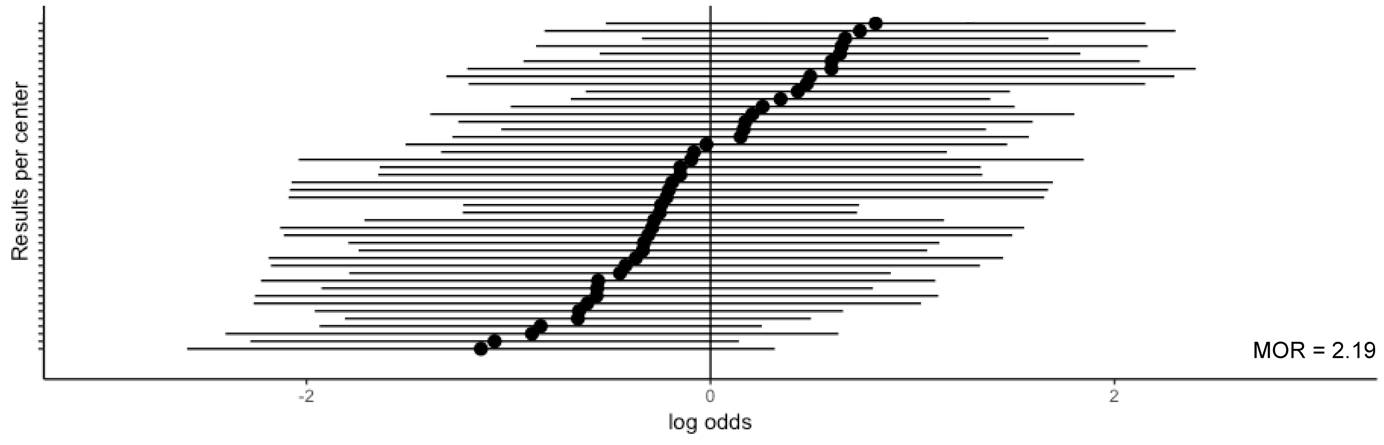
Supplemental Table 12. CT characteristics, surgical motivations and outcome of patients who received secondary decompressive craniectomy**

| **Characteristics** | **Treatment status (n = 336)** | |
| --- | --- | --- |
|  | Primary DC (n = 91) | Craniotomy (n = 245) |
| n (no. secondary decompressive craniectomy) | 14 (15) | 12 (5) ^a^ |
| CT ASDH (%) |  |  |
| Small | 4 (29) | 7 (70) |
| Large | 7 (50) | 3 (30) |
| CT contusion (%) |  |  |
| Small | 6 (43) | 3 (30) |
| Large | 4 (29) | 1 (10) |
| CT SAH (%) |  |  |
| Basal | 0 (0) | 2 (20) |
| Cortical | 1 (7) | 3 (30) |
| Basal and cortical | 3 (21) | 1 (10) |
| CT ischemia (multiple territories) | 0 (0) | 2 (29) |
| CT basal cisterns absent/compressed (%) | 5 (36) | 3 (30) |
| CT midline shift measure (median [IQR]) | 10 [7, 15] | 9 [6, 15] |
| Motivation of surgery |  |  |
| Emergency/life saving | 3 (21) | 1 (10) |
| Clinical deterioration | 3 (21) | 1 (10) |
| Mass effect on CT | 1 (7) | 3 (30) |
| Radiological progression | 3 (21) | 1 (10) |
| (Suspicion of) raised ICP | 4 (29) | 5 (50) |
| In-hospital mortality (%) | 2 (14) | 2 (17) |
| GOSE 6-months (%) |  |  |
| 1=Dead | 4 (29) | 3 (25) |
| 2=Vegetative state/3=Lower severe disability | 5 (36) | 6 (50) |
| 4=Upper severe disability | 1 (7) | 1 (8) |
| 5=Lower moderate disability | 2 (14) | 0 (0) |
| 6=Upper moderate disability | 0 (0) | 0 (0) |
| 7=Lower good recovery | 0 (0) | 1 (8) |
| 8=Upper good recovery | 0 (0) | 0 (0) |
| QOLIBRI (median [IQR]) at 6 months ^b^ | 60 (Na) | 64 (55, 74) |

Abbreviation: ASDH, acute subdural hematoma; DC, decompressive craniectomy; GOSE, Glasgow Outcome Scale Extended; ICP, intracranial pressure; IQR, interquartile range; Na, Not applicable; QOLIBRI, Quality of Life after Brain Injury Scale; SAH, subarachnoid hemorrhage.

^a^ CT outcome data missing for two patients.

^b^ QOLIBRI is a standardized health specific quality of life measure specifically designed for and validated in outcome assesment in patients with brain injury. It is a numerical scale with scores ranging from 0 to 100, with higher scores indicating a better quality of life. The score was available for 1 patient of the primary DC group and 3 patients of the craniotomy group.

**References**

1. Stukel TA, Fisher ES, Wennberg DE, Alter DA, Gottlieb DJ, Vermeulen MJ. Analysis of observational studies in the presence of treatment selection bias: effects of invasive cardiac management on AMI survival using propensity score and instrumental variable methods. *Jama.* 2007;297(3):278-285.

2. Brookhart MA, Rassen JA, Schneeweiss S. Instrumental variable methods in comparative safety and effectiveness research. *Pharmacoepidemiol Drug Saf.* 2010;19(6):537-554.

3. Cnossen MC, van Essen TA, Ceyisakar IE, et al. Adjusting for confounding by indication in observational studies: a case study in traumatic brain injury. *Clin Epidemiol.* 2018;10:841-852.

4. Davies NM, Smith GD, Windmeijer F, Martin RM. Issues in the reporting and conduct of instrumental variable studies: a systematic review. *Epidemiology.* 2013;24(3):363-369.

5. Van Essen TA, Volovici V, Cnossen MC, et al. Comparative effectiveness of surgery in traumatic acute subdural and intracerebral haematoma: study protocol for a prospective observational study within CENTER-TBI and Net-QuRe. *BMJ Open.* 2019;9(10):e033513.

6. van Essen TA, den Boogert HF, Cnossen MC, et al. Variation in neurosurgical management of traumatic brain injury: a survey in 68 centers participating in the CENTER-TBI study. *Acta Neurochir (Wien).* 2019;161(3):435-449.
