# Supplementary material for: Comparative effectiveness of decompressive craniectomy versus craniotomy for traumatic acute subdural hematoma (CENTER-TBI): an observational cohort study
Source: eClinicalMedicine. 2023 Aug 9;63:102161. doi: 10.1016/j.eclinm.2023.102161 (PMC10432786; doi:10.1016/j.eclinm.2023.102161)
Supplement: CENTER-TBI group members [file mmc2.docx]

| **First names** | **Surnames** |
| --- | --- |
| Cecilia | Åkerlund |
| Krisztina | Amrein |
| Nada | Andelic |
| Lasse | Andreassen |
| Audny | Anke |
| Anna | Antoni |
| Gérard | Audibert |
| Philippe | Azouvi |
| Maria Luisa | Azzolini |
| Ronald | Bartels |
| Pál | Barzó |
| Romuald | Beauvais |
| Ronny | Beer |
| Bo-Michael | Bellander |
| Antonio | Belli |
| Habib | Benali |
| Maurizio | Berardino |
| Luigi | Beretta |
| Morten | Blaabjerg |
| Peter | Bragge |
| Alexandra | Brazinova |
| Vibeke | Brinck |
| Joanne | Brooker |
| Camilla | Brorsson |
| Andras | Buki |
| Monika | Bullinger |
| Manuel | Cabeleira |
| Alessio | Caccioppola |
| Emiliana | Calappi |
| Maria Rosa | Calvi |
| Peter | Cameron |
| Guillermo | Carbayo Lozano |
| Marco | Carbonara |
| Ana M. | Castaño-León |
| Simona | Cavallo |
| Giorgio | Chevallard |
| Arturo | Chieregato |
| Giuseppe | Citerio |
| Hans | Clusmann |
| Mark Steven | Coburn |
| Jonathan | Coles |
| Jamie D. | Cooper |
| Marta | Correia |
| Amra | Čović |
| Nicola | Curry |
| Endre | Czeiter |
| Marek | Czosnyka |
| Claire | Dahyot-Fizelier |
| Paul | Dark |
| Helen | Dawes |
| Véronique | De Keyser |
| Vincent | Degos |
| Francesco | Della Corte |
| Hugo | den Boogert |
| Bart | Depreitere |
| Đula | Đilvesi |
| Abhishek | Dixit |
| Emma | Donoghue |
| Jens | Dreier |
| Guy-Loup | Dulière |
| Ari | Ercole |
| Patrick | Esser |
| Erzsébet | Ezer |
| Martin | Fabricius |
| Valery L. | Feigin |
| Kelly | Foks |
| Shirin | Frisvold |
| Alex | Furmanov |
| Pablo | Gagliardo |
| Damien | Galanaud |
| Dashiell | Gantner |
| Guoyi | Gao |
| Pradeep | George |
| Alexandre | Ghuysen |
| Lelde | Giga |
| Ben | Glocker |
| Jagoš | Golubović |
| Pedro A. | Gomez |
| Johannes | Gratz |
| Benjamin | Gravesteijn |
| Francesca | Grossi |
| Russell L. | Gruen |
| Deepak | Gupta |
| Juanita A. | Haagsma |
| Iain | Haitsma |
| Raimund | Helbok |
| Eirik | Helseth |
| Lindsay | Horton |
| Jilske | Huijben |
| Peter J. | Hutchinson |
| Bram | Jacobs |
| Stefan | Jankowski |
| Mike | Jarrett |
| Ji-yao | Jiang |
| Faye | Johnson |
| Kelly | Jones |
| Mladen | Karan |
| Angelos G. | Kolias |
| Erwin | Kompanje |
| Daniel | Kondziella |
| Evgenios | Kornaropoulos |
| Lars-Owe | Koskinen |
| Noémi | Kovács |
| Alfonso | Lagares |
| Linda | Lanyon |
| Steven | Laureys |
| Fiona | Lecky |
| Didier | Ledoux |
| Rolf | Lefering |
| Valerie | Legrand |
| Aurelie | Lejeune |
| Leon | Levi |
| Roger | Lightfoot |
| Hester | Lingsma |
| Andrew I.R. | Maas |
| Marc | Maegele |
| Marek | Majdan |
| Alex | Manara |
| Geoffrey | Manley |
| Hugues | Maréchal |
| Costanza | Martino |
| Julia | Mattern |
| Catherine | McMahon |
| Béla | Melegh |
| David | Menon |
| Tomas | Menovsky |
| Ana | Mikolic |
| Benoit | Misset |
| Visakh | Muraleedharan |
| Lynnette | Murray |
| Nandesh | Nair |
| Ancuta | Negru |
| David | Nelson |
| Virginia | Newcombe |
| Daan | Nieboer |
| József | Nyirádi |
| Matej | Oresic |
| Fabrizio | Ortolano |
| Olubukola | Otesile |
| Aarno | Palotie |
| Paul M. | Parizel |
| Jean-François | Payen |
| Natascha | Perera |
| Vincent | Perlbarg |
| Paolo | Persona |
| Wilco | Peul |
| Anna | Piippo-Karjalainen |
| Matti | Pirinen |
| Dana | Pisica |
| Horia | Ples |
| Suzanne | Polinder |
| Inigo | Pomposo |
| Jussi P. | Posti |
| Louis | Puybasset |
| Andreea | Rădoi |
| Arminas | Ragauskas |
| Rahul | Raj |
| Malinka | Rambadagalla |
| Veronika | Rehorčíková |
| Isabel | Retel Helmrich |
| Jonathan | Rhodes |
| Sylvia | Richardson |
| Sophie | Richter |
| Samuli | Ripatti |
| Saulius | Rocka |
| Cecilie | Roe |
| Olav | Roise |
| Jonathan | Rosand |
| Jeffrey | Rosenfeld |
| Christina | Rosenlund |
| Guy | Rosenthal |
| Rolf | Rossaint |
| Sandra | Rossi |
| Daniel | Rueckert |
| Martin | Rusnák |
| Juan | Sahuquillo |
| Oliver | Sakowitz |
| Renan | Sanchez-Porras |
| Janos | Sandor |
| Nadine | Schäfer |
| Silke | Schmidt |
| Herbert | Schoechl |
| Guus | Schoonman |
| Rico Frederik | Schou |
| Elisabeth | Schwendenwein |
| Charlie | Sewalt |
| Toril | Skandsen |
| Peter | Smielewski |
| Abayomi | Sorinola |
| Emmanuel | Stamatakis |
| Simon | Stanworth |
| Ana | Kowark |
| Robert | Stevens |
| William | Stewart |
| Ewout W. | Steyerberg |
| Nino | Stocchetti |
| Nina | Sundström |
| Riikka | Takala |
| Viktória | Tamás |
| Tomas | Tamosuitis |
| Mark Steven | Taylor |
| Braden | Te Ao |
| Olli | Tenovuo |
| Alice | Theadom |
| Matt | Thomas |
| Dick | Tibboel |
| Marjolijn | Timmers |
| Christos | Tolias |
| Tony | Trapani |
| Cristina Maria | Tudora |
| Andreas | Unterberg |
| Peter | Vajkoczy |
| Egils | Valeinis |
| Shirley | Vallance |
| Zoltán | Vámos |
| Mathieu | Van der Jagt |
| Joukje | van der Naalt |
| Gregory | Van der Steen |
| Jeroen T.J.M. | van Dijck |
| Thomas A. | van Essen |
| Wim | Van Hecke |
| Caroline | van Heugten |
| Dominique | Van Praag |
| Ernest | Van Veen |
| Roel | van Wijk |
| Thijs | Vande Vyvere |
| Alessia | Vargiolu |
| Emmanuel | Vega |
| Kimberley | Velt |
| Jan | Verheyden |
| Paul M. | Vespa |
| Anne | Vik |
| Rimantas | Vilcinis |
| Victor | Volovici |
| Nicole | von Steinbüchel |
| Daphne | Voormolen |
| Petar | Vulekovic |
| Kevin K.W. | Wang |
| Eveline | Wiegers |
| Guy | Williams |
| Lindsay | Wilson |
| Stefan | Winzeck |
| Stefan | Wolf |
| Zhihui | Yang |
| Peter | Ylén |
| Alexander | Younsi |
| Frederick A. | Zeiler |
| Agate | Ziverte |
| Tommaso | Zoerle |
